# Advancing CAR-based cell therapies for solid tumours: challenges, therapeutic strategies, and perspectives

**DOI:** 10.1186/s12943-025-02386-8

**Published:** 2025-07-07

**Authors:** Sarkar Sardar Azeez, Raya Kh. Yashooa, Shukur Wasman Smail, Abbas Salihi, Azhin Saber Ali, Sami Mamand, Christer Janson

**Affiliations:** 1 Department of Medical Laboratory Technology, Soran Technical College, Erbil Polytechnic University, Erbil, Kurdistan Region 44008 Iraq; 2 Department of Biology, College of Education for Pure Sciences, University of Al-Hamdaniya, Mosul, 41002 Iraq; 3 College of Pharmacy, Cihan University-Erbil, Erbil, Kurdistan Region Iraq; 4 Department of Biology, College of Science, Salahaddin University-Erbil, Erbil, Kurdistan Region Iraq; 5 Center of Research and Strategic Studies, Lebanese French University, Kurdistan Region, Erbil, Kurdistan Region 44002 Iraq; 6 Department of Medical Laboratory Technology, Shaqlawa Technical College, Erbil Polytechnic University, Erbil, Kurdistan Region Iraq; 7 Department of Immunology, University of Toronto, Toronto, ON Canada; 8 Department of Medical Science, Respiratory Medicine, and Allergology, Uppsala University and University Hospital, Uppsala, Sweden

**Keywords:** CAR-T cells, CAR-NK cells, CAR-Macrophages, Combination therapy, Challenges, Optimisation Strategies, Solid tumours

## Abstract

Chimeric antigen receptor-cell therapies have demonstrated remarkable success in haematological malignancies but face significant hurdles in solid tumours. The hostile tumour microenvironment, antigen heterogeneity, limited tumour infiltration, and CAR-cell exhaustion contribute to reduced efficacy. Additionally, toxicity, off-target effects, and manufacturing challenges limit widespread clinical adoption. Overcoming these barriers requires a multifaceted approach that enhances CAR-cell persistence, trafficking, and tumour-specific targeting. Recent advancements in alternative cellular therapies, such as CAR-natural killer cells, CAR-macrophages, gamma delta CAR-T cells, and CAR-natural killer T cells, provide promising avenues for improving efficacy. These strategies leverage distinct immune cell properties to enhance tumour recognition and persistence. Furthermore, combination therapies, including chemotherapy, radiotherapy, antibodies, small molecule inhibitors, cancer vaccines, oncolytic viruses, and multi-CAR cell combination therapy, offer synergistic potential by modulating the TME and improving CAR-cell functionality. This review explores the challenges of CAR-based cellular therapies in solid tumours and highlights emerging strategies to overcome therapeutic limitations. By integrating novel cellular platforms and combination approaches, we seek to provide insights into optimising CAR-cell therapies for durable responses in solid malignancies.

## Introduction

**Fig. 1:**
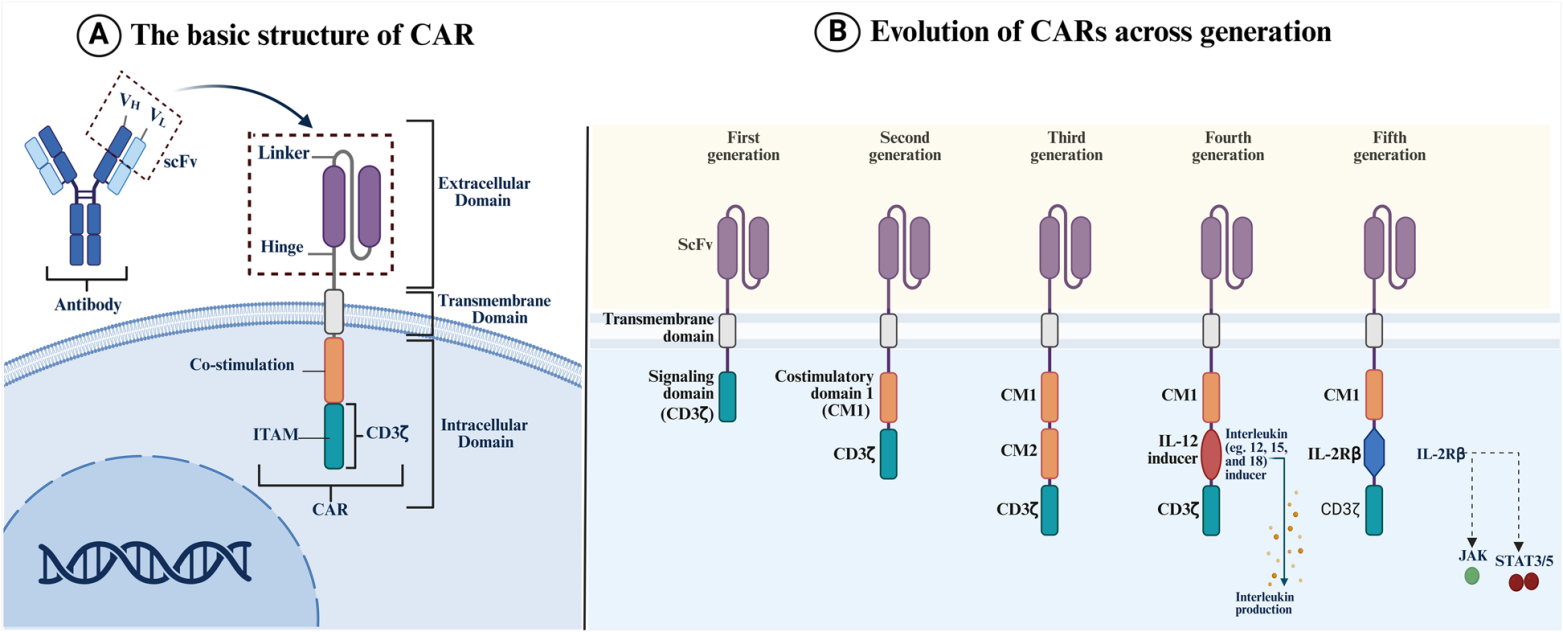
The basic structure and evolution of CARs across generations. **A** Schematic representation of the canonical CAR architecture, consisting of an extracellular single-chain variable fragment (scFv) derived from tumour antigen-specific antibodies for antigen recognition, a hinge or spacer region for conformational flexibility, a transmembrane domain (commonly derived from CD4, CD8α, or CD28) for membrane anchoring, and an intracellular signalling module. The intracellular domain includes the CD3ζ chain, containing immunoreceptor tyrosine-based activation motifs (ITAMs) for T-cell activation, often in combination with co-stimulatory domains [5, 6]. **B** Outlines the evolution of CAR generations. First-generation CARs consist solely of the CD3ζ intracellular signalling domain, which initiates T-cell activation but lacks co-stimulatory signals necessary for robust in vivo function [6]. Second-generation CARs introduce a single co-stimulatory domain—most commonly CD28 or 4-1BB—enhancing T-cell expansion, persistence, and anti-tumour efficacy. CD28-based CARs drive rapid effector responses, while 4-1BB confers superior metabolic fitness and durability [7, 8]. Third-generation CARs incorporate two co-stimulatory domains (e.g., CD28 + 4-1BB), offering synergistic enhancement of cytokine production and cytotoxicity [9, 10]. Fourth-generation CARs, or TRUCKs (T cells Redirected for Universal Cytokine-mediated Killing), include inducible cytokine-expression modules (e.g., IL-12, IL-15, IL-18) that modulate the immunosuppressive TME and improve local immune activation [11]. Fifth-generation CARs build on second-generation frameworks by integrating a truncated IL-2Rβ chain with a STAT3/5-binding motif, enabling direct activation of the JAK–STAT signalling cascade to enhance T-cell proliferation, memory differentiation, and long-term anti-tumour responses [12]

Cancer remains one of the leading causes of mortality worldwide, with approximately 9.7 million deaths recorded in 2022, underscoring the need for innovative treatment approaches [1]. Cancer immunotherapy has revolutionised cancer treatment by leveraging the immune system's ability to identify and destroy malignant cells [2]. The development of chimeric antigen receptor (CAR)-T cell therapy marks a milestone in cancer immunotherapy (The basic structure of CAR is shown in Fig. 1). CAR-T cells are genetically engineered immune cells repurposed to specifically target tumour antigens expressed on malignant cells by expressing modified T-cell receptors, thereby bypassing the need for antigen presentation through major histocompatibility complexes (MHCs) [3]. This precise targeting has given the CAR-T cells a potent anti-tumour response, particularly in cancers characterised by well-defined surface antigens [4].

The clinical success of CAR T-cell therapy was first demonstrated in haematological malignancies, where CD19-targeted CAR-T cells revolutionised the treatment of relapsed/refractory (R/R) B-cell malignancies [13]. Since the FDA approval of the first CAR T-cell therapy (tisagenlecleucel) in 2017, multiple CAR T-cell products have been approved for acute lymphoblastic leukaemia (ALL), diffuse large B-cell lymphoma (DLBCL), mantle cell lymphoma (MCL), and multiple myeloma (MM)[14–20]. These therapies have shown high response rates, prolonged remission in some patients, and durable disease control, establishing CAR-T cells as a transformative therapeutic modality in haematological cancers. Yet, several challenges persist, including adverse effects such as Immune Effector Cell-Associated Neurotoxicity Syndrome (ICANS) and cytokine release syndrome (CRS), as well as patient relapse within the first year post-infusion [21].

Unlike haematological cancers, solid tumours present a more complex microenvironment characterised by heterogeneous antigen expression, an immunosuppressive tumour microenvironment (TME), and physical barriers such as a dense extracellular matrix (ECM), abnormal vasculature, hypoxia, and metabolic stress. These factors have impaired CAR-T cell function and contributed to therapeutic resistance and relapse [22–25]. To date, no CAR T-cell therapy has received FDA approval for solid tumours [26]. Early clinical trials employing first-generation CAR-T cells targeting antigens such as HER2 and CAIX in glioblastoma and renal cell carcinoma, respectively, revealed limited therapeutic benefit, primarily due to poor in vivo persistence and off-tumour toxicities [27–29]. Second-generation CAR-T cells demonstrated enhanced antitumour activity in preclinical models. However, clinical trials have shown only modest response rates and frequent dose-limiting toxicities [30, 31]. Similarly, third-generation CAR-T cells with improved persistence through dual co-stimulatory signal engineering yielded inconsistent clinical responses and increased immunotoxicity [32, 33]. Fourth- and fifth-generation CARs, engineered with cytokine secretion and cytokine-integrated signalling domains, respectively, have shown improved tumour infiltration and immune modulation capabilities in early trials, yet their use has been constrained by systemic toxicity and manufacturing complexities [11, 34, 35] (The basic structure and evolution of CARs across generations is illustrated in Fig. 1). Consequently, while significant advances have been made in CAR design for solid tumours, clinical translation remains an ongoing challenge.

Alternative CAR-based approaches, including CAR-natural killer (CAR-NK) cells, CAR-macrophages (CAR-Ms), CAR-gamma delta T cells (γδ CAR-T cells), and CAR-natural killer T cells (CAR-NKT) cell therapies, are being actively developed. These strategies leverage distinct immune mechanisms to enhance tumour targeting, overcome tumour resistance, improve cell persistence, and enable the use of universal cell sources for production; however, they also face significant hurdles, such as limited persistence (CAR-NK cells), functional suppression within the TME, and off-target effects [36–38].

This review aims to comprehensively analyse ongoing and future advancements in CAR-cell therapies, primarily focusing on CAR T-cell therapy for solid tumours. It examines the evolution of CAR constructs, key challenges, and therapeutic limitations. Importantly, potential strategies to enhance CAR-T cells' efficacy, safety, and feasibility are discussed. Additionally, emerging CAR-based therapies, such as CAR-NK cells, CAR-Ms, γδ CAR-T, and CAR-NKT cells, are considered promising alternatives. Furthermore, we also explore the potential combination approaches, including chemotherapy, radiotherapy, antibodies, small molecule inhibitors, cancer vaccines, oncolytic viruses, and multi-car-cell (CAR-T, NK, and Ms) combinations that may improve CAR-cell persistence and tumour infiltration, ultimately expanding the therapeutic potential of CAR-based immunotherapies.

## Challenges and optimisation strategies of CAR-T cell therapy in solid tumours

### The tumour microenvironment

**Fig. 2:**
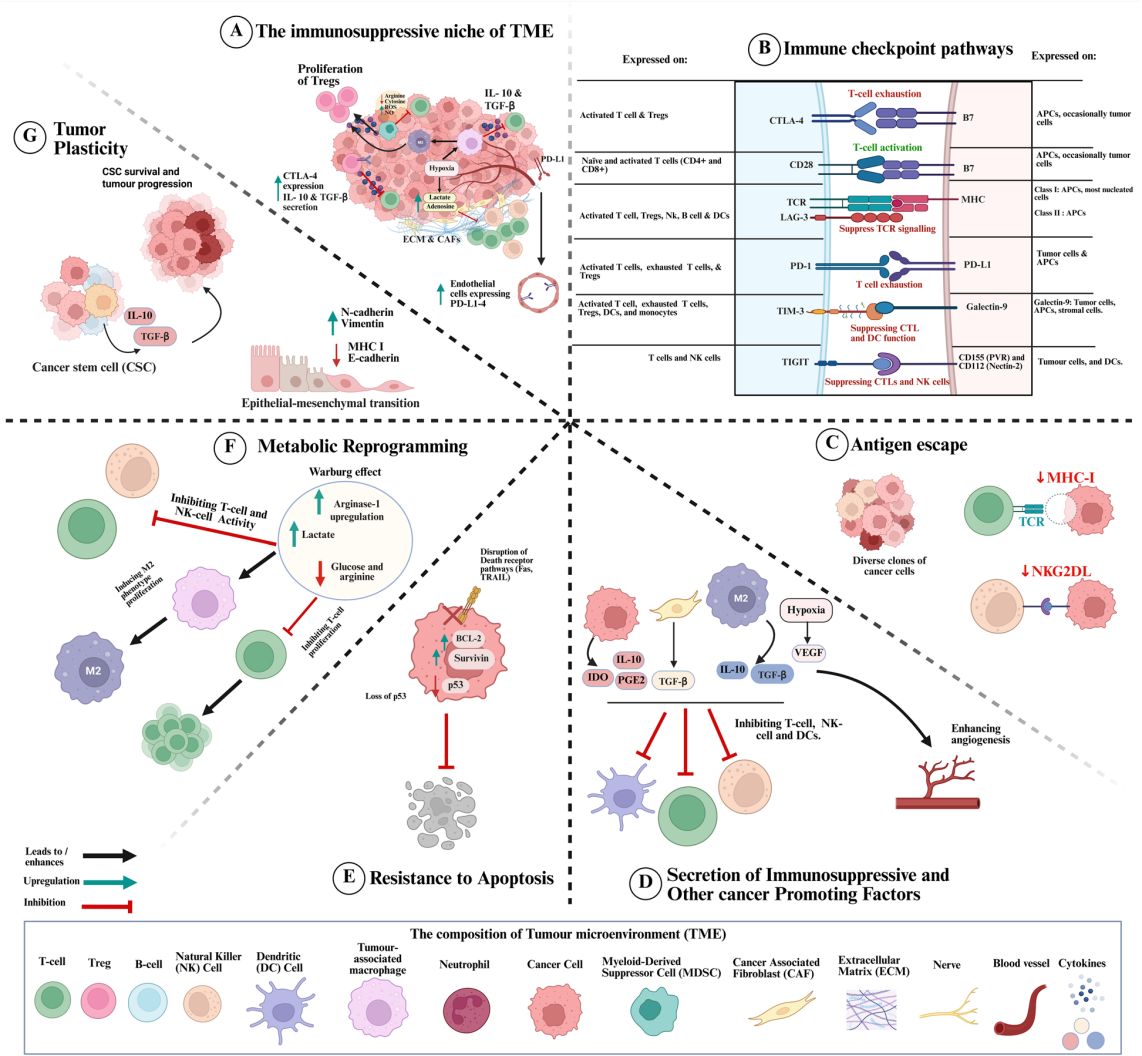
This figure provides a comprehensive overview of the mechanisms that enable tumours to evade immune surveillance and resist immune-mediated destruction, forming a critical framework for understanding immune resistance in cancer. **A** The TME plays a central role in promoting immunosuppression through various interconnected processes. Tumours create an immunosuppressive niche by promoting the expansion of Tregs, MDSCs, and CAFs, which secrete inhibitory cytokines like TGF-β and IL-10 to suppress cytotoxic immune responses. **B** Immune checkpoint pathways, including PD-L1, CTLA-4, TIM-3, and LAG-3, further contribute to immune evasion by inducing T-cell exhaustion and impairing CTL responses. **C** In addition, tumours escape immune recognition by downregulating major MHC-I molecules and NK-cell-activating ligands (e.g., NKG2DL), reducing antigen presentation and cytolytic activity. **D** Beyond direct immune modulation, tumour cells and stromal components secrete immunosuppressive and pro-tumourigenic factors such as IL-10, TGF-β, PGE2, and VEGF, which inhibit T-cell and NK-cell function while promoting angiogenesis and disease progression. **E** Resistance to apoptosis is another critical factor, where genetic and epigenetic alterations, including p53 loss and overexpression of survival proteins like BCL-2 and survivin, disrupt cell death pathways, making tumour cells resistant to immune-mediated killing. **F** Furthermore, metabolic reprogramming within the TME, such as the Warburg effect and increased lactate production, leads to an immunosuppressive metabolic landscape that further inhibits T-cell and NK-cell function. **G** Lastly, tumour plasticity, driven by EMT and cancer stem cell (CSC) survival, enhances immune evasion, therapy resistance, and metastatic potential. Together, these mechanisms create a formidable barrier to effective immunotherapy, highlighting the need for novel strategies to counteract immune resistance in cancer treatment

The TME presents a major obstacle to CAR-T cell efficacy in solid tumours by fostering an immunosuppressive niche that limits T cell function, infiltration, and persistence (Fig. 2) [39]. The TME is composed of various immunosuppressive cells, including tumour-associated macrophages (TAMs), myeloid-derived suppressor cells (MDSCs), regulatory T cells (Tregs), endothelial cells, and cancer-associated fibroblasts (CAFs), all embedded within a dense ECM [40, 41]. TAMs, particularly those polarised to the M2 phenotype, secrete interleukin-10 (IL-10) and transforming growth factor-β (TGF-β), which suppress cytotoxic T lymphocytes (CTLs) and promote angiogenesis [42]. MDSCs further contribute to immune suppression by depleting essential nutrients such as arginine and cysteine, producing reactive oxygen species (ROS) and nitric oxide (NO), and secreting IL-10 and TGF-β, which enhance Treg expansion [43–45]. Regulatory T cells (Tregs) also play a central role in immune evasion by expressing cytotoxic T-lymphocyte-associated protein 4 (CTLA-4) and secreting IL-10 and TGF-β, which inhibit CTLs and NK cells [46]. The recruitment of Tregs to the TME is facilitated by chemokines such as CCL22 and hypoxia, further reinforcing immune suppression [47]. Additionally, the ECM, primarily structured by CAFs, acts as a physical barrier to immune infiltration while also secreting TGF-β to suppress anti-tumour immunity [48]. Endothelial cells contribute to tumour immune evasion by inducing vascular abnormalities that lead to hypoxia and impair immune cell infiltration [49]. They also express programmed death-ligand 1 (PD-L1), which directly suppresses T cell activity and enhances interactions with immunosuppressive MDSCs and Tregs [50].

Moreover, tumour cells exhibit distinct intrinsic resistance mechanisms to CAR-T cell-mediated killing, varying between solid and haematological malignancies. For instance, Larson et al. showed that IFNγ receptor signalling is required for CAR-T cells to eliminate solid tumour cells but not liquid tumour cells. This highlights distinct resistance mechanisms in solid tumours [51].

The metabolic landscape of the TME further limits CAR-T cell efficacy. Hypoxia, driven by rapid tumour growth and inadequate vascularisation, stabilises hypoxia-inducible factor 1-α (HIF-1α), which upregulates vascular endothelial growth factor (VEGF) and promotes angiogenesis while also enhancing immunosuppression through increased adenosine and lactate production [52, 53]. High lactate concentrations impair CTL and NK cell function, polarise TAMS toward the immunosuppressive M2 phenotype, and further increase IL-10 and TGF-β secretion [54–56]. Tumour cells also compete aggressively for nutrients such as glucose and arginine, depriving immune cells of essential resources needed for activation [57]. The upregulation of metabolic enzymes like arginase-1 exacerbates this deprivation by depleting arginine, inhibiting T-cell proliferation, and cytokine production [58]. Altogether, this complex interplay of immunoevasive strategies enables malignant cells to circumvent immune surveillance and therapeutic interventions.

#### Overcoming immunosuppression within the tumour microenvironment

Several therapeutic strategies are being explored to optimise CAR-T cell efficacy in solid tumours (Fig. 4A) [59]. One promising approach is engineering CAR-T cells to express cytokines or genetic modifications that reverse the immunosuppressive nature of the TME. For instance, armoured CAR-T cells such as TRUCKs are engineered to secrete various cytokines such as interleukin-15 (IL-15) and interleukin-21 (IL-21). IL-15 promotes the expansion and persistence of CAR-T cells, leading to improved anti-tumour activity [34]. Similarly, IL-21 enhances the generation of less differentiated T-cell subsets, which are associated with improved persistence and anti-tumour activity [60]. Moreover, IL-12 repolarises M2 macrophages into a pro-inflammatory M1 phenotype and enhances T cell persistence [61]. However, a significant challenge in CAR-T cell therapy is CRS, which can range from mild flu-like symptoms to severe, life-threatening conditions, including capillary leak syndrome and multi-organ dysfunction [62]. By designing CAR-T cells with controlled cytokine production or responsiveness, researchers aim to enhance anti-tumour efficacy while minimising adverse effects like CRS. These approaches include engineering self-regulating CAR-T cells capable of modulating cytokine release in response to the TME [63].

Eliminating or functionally altering immunosuppressive cells like TAMs, MDSCs, and Tregs improves CAR-T efficacy. Inhibiting the colony-stimulating factor 1 receptor (CSF1R) can effectively deplete M2 macrophages and reprogram them toward a pro-inflammatory phenotype, thereby enhancing anti-tumour immunity [64]. Additionally, MDSCs secrete arginase and indoleamine 2,3-dioxygenase (IDO), which degrade arginine and tryptophan, key metabolites required for T cell activation [65]. Blocking these enzymes restores immune cell function and promotes T-cell-mediated responses [66, 67].

Addressing hypoxia and metabolic constraints is crucial for enhancing CAR-T cell metabolic fitness and resistance to hypoxia-induced immunosuppression. One strategy involves engineering CAR-T cells to resist hypoxia-inducible factor 1-α (HIF-1α) signalling, thereby maintaining their function in low-oxygen environments [68, 69]. Additionally, tumour MDSC cells release adenosine, suppressing T-cell activity through A2A receptors [70]. Modifying CAR-T cells to be A2A receptor-deficient enables them to sustain their function within the TME [71]. Moreover, enhancing oxidative phosphorylation (OXPHOS) in CAR-T cells allows them to function effectively even in glucose-deprived conditions, conferring resistance to metabolic stress [72, 73].

Effective immunity against tumours relies on T-cell fatty acid uptake and energy production. TAGLN2 facilitates lipid uptake and mitochondrial respiration in CD8+ T cells by interacting with FABP5 [74]. Tumour-induced ER stress suppresses TAGLN2, impairing T cell function in ovarian cancer. Restoring TAGLN2 expression enhances lipid uptake, mitochondrial function, and cytotoxicity. CAR-T cells overexpressing TAGLN2 bypass ER stress and show improved therapeutic efficacy in ovarian cancer models. This highlights the potential of the TAGLN2-FABP5 axis to enhance cellular immunotherapy in solid tumours [75]. The hostile TME remains a significant challenge for CAR-T therapy in solid tumours. However, emerging strategies, including armoured CAR-T cells, eliminating or altering immunosuppressive cells in the TME, and metabolic reprogramming, hold great potential to improve CAR-T efficacy.

### Limited tumour infiltration and homing

A significant barrier to the success of CAR-T cell therapy in solid tumours is the inefficient infiltration of CAR-T cells into the tumour mass (Fig. 2). Unlike haematological malignancies, where CAR-T cells can circulate freely in the bloodstream and directly interact with malignant cells, solid tumours present formidable physical and biochemical obstacles that prevent efficient T-cell trafficking and infiltration [76]. In solid tumours, angiogenic signalling is dysregulated, resulting in blood vessels that are structurally abnormal and functionally inefficient. These vessels are characteristically tortuous, dilated, and hyperpermeable, leading to uneven blood flow and elevated interstitial fluid pressure. Such conditions hinder the transvascular migration of immune cells by reducing the expression of adhesion molecules on the endothelium and creating a physical barrier that impedes extravasation, thereby limiting the efficacy of immunotherapeutic interventions, including CAR-T cell therapies [77]. Additionally, the aberrant vasculature often leads to hypoxia and nutrient deprivation regions, further hindering immune cell trafficking [49].

Solid tumours also manipulate chemokine signalling to create an immunosuppressive landscape that misguides or excludes CAR-T cells [78].

Chemokines are key mediators of T-cell migration, but tumours frequently downregulate the expression of chemokines required for T-cell homing, such as CXCL9 and CXCL10, while upregulating signals that preferentially attract immunosuppressive cells, such as Tregs and MDSCs [79]. For example, endothelial cells in the TME often overexpress CCL22, which preferentially recruits Tregs rather than effector T cells [80]. A mismatch between chemokine receptors expressed on CAR-T cells and the chemokines secreted by tumours results in poor T cell migration into the TME [81]. Furthermore, they upregulate immune checkpoint molecules such as programmed death-ligand 1 (PD-L1), further impairing CAR-T cell function (Fig. 2B) [82, 83]. Without appropriate chemokine signalling, CAR-T cells remain sequestered in circulation or peripheral tissues, failing to reach and accumulate at tumour sites.

Another primary impediment is the dense ECM, composed of collagen, fibronectin, and glycosaminoglycans, which physically restricts T-cell migration into the tumour core [84]. This matrix is largely shaped by CAFs, further remodels the ECM, and secretes factors such as TGF-β to suppress immune infiltration [85].

#### Enhancing CAR-T cell trafficking and tumour homing

Local administration of CAR-T cells is a promising strategy to overcome limited tumour infiltration and homing (Fig. 4B). This approach confines CAR-T cells to the TME, thereby enhancing their accumulation at the target site and reducing interactions with normal tissues. In a study, intrapleural delivery of anti-mesothelin CAR-T cells has effectively eliminated pleural tumours at substantially lower doses compared to systemic administration while eliciting durable systemic anti-tumour immunity through migration to adjacent tumour regions [86]. In addition, regional delivery in brain metastasis models has demonstrated that direct intracranial injection leads to earlier and increased tumour localisation compared to intracerebroventricular routes, resulting in improved therapeutic outcomes despite similar overall efficacy [87].

Another transformative strategy addresses chemokine mismatches by engineering CAR-T cells to express chemokine receptors that align with tumour-secreted chemokines, enhancing their ability to home the tumour (Tables 1 and 2). For example, CAR-T cells expressing CXCR2 or CCR5 have demonstrated improved migration toward tumours that produce CXCL1, CXCL8, or CCL5 [88–90]. In addition to chemokine receptor modifications, CAR-T cells can be enhanced with integrins, adhesion molecules that improve interactions between T cells and the tumour endothelium. These modifications facilitate the extravasation and retention of CAR-T cells within the TME, increasing the duration of immune cell engagement with tumour cells [91]. Tables 1 and 2 summarise pro- and anti-tumour chemokines and their receptors across various cell types and tumours, identifying targets to improve CAR-T-cell tumour homing. Targeting pro-tumour chemokines may also block the recruitment of immunosuppressive cells. For detailed insights, see Mollica Poeta et al. (2019), Chow and Luster (2014), and Kohli et al. (2022) [79, 92, 93].

**Table 1:** Pro-tumour chemokines (recruit immunosuppressive cells and prevent immune cell infiltration)

Chemokine	Receptor	Pro-tumour function	References
CCL5 (RANTES)	CCR5	Attracts Tregs and MDSCs, promoting immune evasion	[94, 95]
CCL17	CCR4	Recruits Tregs, inhibiting effector T-cell responses	[96]
CCL22	CCR4	Attracts Tregs to suppress anti-tumour immune responses	[97]
CXCL12 (SDF-1)	CXCR4	Recruits TAMs and MDSCs, regulates and maintains cancer stem cells	[98, 99]
CXCL8 (IL-8)	CXCR1, CXCR2	Recruits neutrophils and TAMs, promotes angiogenesis, and immune suppression	[100]
CXCL1	CXCR2	Supports tumour growth and angiogenesis; promotes MDSC accumulation	[101]
CXCL5	CXCR2	Attracts MDSCs; promotes neutrophil recruitment and immune evasion	[102, 103]

**Table 2:** Anti-tumour chemokines (Facilitate immune cell infiltration)

Chemokine	Receptor	Function	References
CXCL9 (MIG)	CXCR3	Attracts CD8 + T cells, Th1 cells for tumour clearance	[104, 105]
CXCL10 (IP-10)	CXCR3	Enhances T-cell infiltration; promotes anti-tumour immunity	[106]
CXCL11 (I-TAC)	CXCR3	Increases CD8 + T-cell homing and tumour infiltration	[107]
CX3CL1 (Fractalkine)	CX3CR1	Attracts CD8 + T cells and NK cells, increasing tumour immune infiltration	[108, 109]
CCL3 (MIP-1α)	CCR1, CCR5	Promotes T-cell and DC recruitment into tumours	[110, 111]
CCL4 (MIP-1β)	CCR5	Enhances CD8 + T-cell responses, increasing tumour clearance	[112]
CXCL16	CXCR6	Attracts CD8 + T cells and NK cells to promote anti-tumour responses	[113, 114]
CCL21	CCR7	Promotes DC and T-cell recruitment, enhancing adaptive immune responses	[115, 116]

Modifying CAR-T cells to degrade the ECM actively creates pathways for infiltration. Engineering CAR-T cells to express heparanase (HPSE), an enzyme that degrades heparan sulphate proteoglycans in the ECM, has enhanced tumour penetration and improved therapeutic efficacy [117].

Another strategy is targeting CAFs, major contributors to ECM stiffness and immune exclusion [118]. CAR-T cells designed to target fibroblast activation protein (FAP), a surface marker highly expressed on CAFs, can selectively deplete these stromal cells, reducing ECM density and enhancing T cell infiltration. FAP inhibitors or FAP-CAR-T cells can further modulate the tumour stroma, allowing for improved access of effector T cells to the tumour core [119, 120].

CAR-T cell infiltration can be enhanced by its combination with radiation therapy or chemotherapy. Radiation therapy, in particular, has been shown to enhance T-cell infiltration by upregulating the expression of adhesion molecules on endothelial cells and increasing the release of inflammatory cytokines that promote immune cell recruitment [121]. Similarly, low-dose chemotherapy can deplete immunosuppressive cells such as MDSCs, creating a more permissive microenvironment for CAR-T cell activity (described in more detail in the following sections) [122]. Overcoming the infiltration challenges of CAR-T therapy in solid tumours requires a multi-faceted approach that combines ECM degradation, stromal targeting, chemokine receptor engineering, adhesion molecule modifications, and adjunctive treatments such as radiation and chemotherapy. These strategies collectively enhance CAR-T homing, migration, and retention within the TME, ultimately improving anti-tumour responses.

### Antigen heterogeneity and loss

One of the most significant challenges limiting the efficacy of CAR-T cell therapy in solid tumours is antigen heterogeneity and loss (Fig. 2C). Unlike haematological malignancies, which often express stable and homogeneous antigens like CD19 or BCMA, solid tumours exhibit considerable antigenic diversity within and between patients, even among cells of a tumour mass [123].

Effective immune recognition of tumours relies on the presentation of tumour-associated antigens (TAAs) and tumour-specific antigens (TSAs) via MHC molecules, a crucial step for CTL activation. Tumours commonly reduce or lose MHC class I expression, impairing the presentation of antigens to CD8 + CTLs and escaping immune detection [124]. This downregulation often occurs through genetic mutations or epigenetic alterations in β2-microglobulin or defects in transporter associated with antigen processing (TAP) proteins that prevent the proper loading of tumour-derived peptides onto MHC class I molecules, thereby rendering the tumour cells invisible to CTLs [125, 126]. Although NK cells can detect "missing self" via absent MHC molecules, tumours counteract by downregulating NKG2D ligands, avoiding NK-mediated lysis [127].

Additionally, intratumoral heterogeneity results in subpopulations of tumour cells with differing antigen expression profiles, making it difficult for single-antigen CAR-T cells to eradicate malignant cells effectively [128, 129]. This is further exacerbated by antigen escape mechanisms, where tumour cells undergo epigenetic modifications or upregulate alternative survival pathways to circumvent immune attack [130]. Consequently, targeting a single TAA often leads to partial tumour clearance, with antigen-negative or low-antigen-expressing cells surviving and driving disease relapse [131].

#### Broadening antigen recognition to overcome tumour heterogeneity

**Fig. 3:**
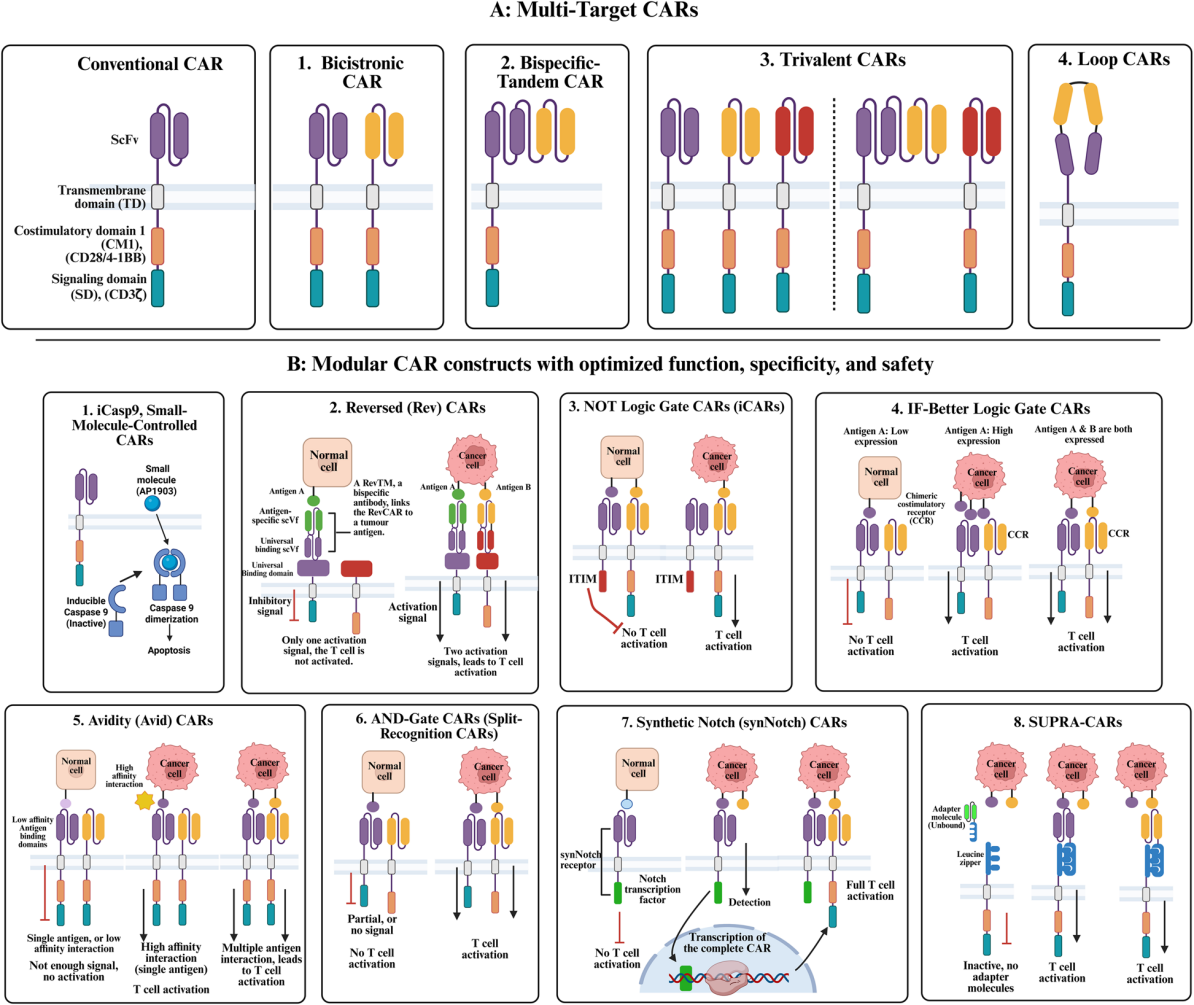
This illustration presents advanced car designs, categorised into multi-target cars and modular car constructs with optimised function, specificity, and safety. **A** The Multi-Target CARs are designed to enhance antigen recognition and tumour targeting by incorporating multiple antigen-binding domains. These include Bicistronic CARs, which express two independent CAR constructs within a single T cell to target distinct antigens; Bispecific-Tandem CARs, which contain a single receptor with dual antigen-binding domains; Trivalent CARs, which incorporate three antigen-binding sites to increase specificity and reduce tumour escape; and Loop CARs, which feature a looped scFv structure to improve antigen-binding flexibility. **B** The Modular CAR Constructs integrate additional regulatory mechanisms to refine CAR-T cell activation, specificity, and safety. iCasp9 Small-Molecule-Controlled CARs include an inducible caspase-9 suicide switch that allows for the controlled depletion of CAR-T cells in case of severe toxicity. Reversed (Rev) CARs require a bispecific bridging molecule for activation, preventing unwanted tonic signalling. NOT Logic Gate CARs (iCARs) integrate inhibitory receptors that suppress activation upon recognising an off-target antigen, reducing damage to healthy tissues. IF-Better Logic Gate CARs introduce an activation threshold that ensures T-cell activation only in response to high antigen expression, preventing reactivity to normal tissues with low antigen levels. Avidity (Avid) CARs require bivalent antigen binding for full activation, preventing responses to cells with low antigen expression. AND-Gate CARs incorporate two separate receptors, where both antigens must be engaged to trigger full T-cell activation, ensuring strict tumour selectivity. Synthetic Notch (synNotch) CARs enable sequential antigen recognition, where binding to a first antigen induces the expression of a second CAR, allowing for delayed and more precise T-cell activation. Lastly, SUPRA-CARs (Split, Universal, and Programmable CARs) utilise a universal receptor system with modular adapter molecules, allowing for real-time reprogramming of antigen specificity without requiring genetic modification of T cells. These next-generation CAR constructs provide enhanced precision, flexibility, and safety in CAR-T cell therapy, making them promising candidates for improved treatment of both haematological malignancies and solid tumours

**Fig. 4:**
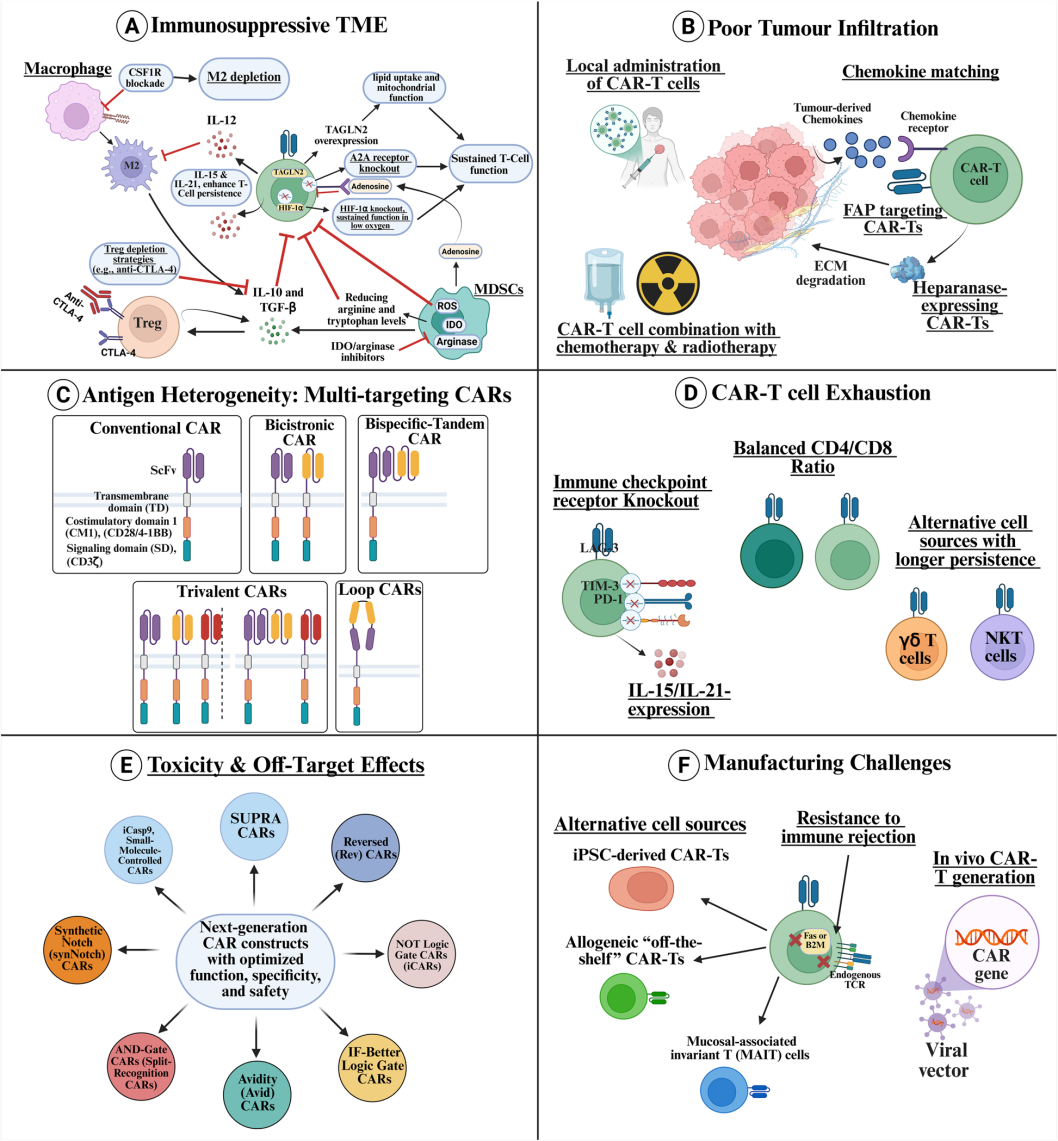
This figure illustrates potential strategies to overcome the challenges of CAR-T cells in solid tumours. Several barriers hinder the efficacy of CAR-T cell therapy in solid malignancies, necessitating novel strategies for optimisation. **A** Immunosuppressive TME: Engineering CAR-T cells with HIF-1α knockout or TAGLN2 overexpression enhances their function in hypoxic conditions. Macrophage reprogramming via CSF1R inhibition and MDSC modulation with IDO/arginase inhibitors restores immune-activating conditions. Treg depletion using anti-CTLA-4 strategies further improves immune response. **B** Poor Tumour Infiltration: Strategies include chemokine receptor expression matching tumour-derived chemokines, ECM degradation via heparanase-expressing CAR-Ts, and combination therapies with chemotherapy and radiotherapy to improve homing and penetration. **C** Antigen Heterogeneity: Multi-target CAR designs such as bicistronic, bispecific-tandem, trivalent, and loop CARs address tumour antigenic variability and escape mechanisms (Refer to Fig. 3). **D** CAR-T Exhaustion: Enhancing persistence through immune checkpoint receptor knockout (LAG-3, PD-1, TIM-3), cytokine modulation (IL-15/IL-21), and alternative cell sources with longer persistence abilities like γδ T and NKT cells. **E** Toxicity & Off-Target Effects: Modular CAR constructs optimise specificity and safety, including small-controlled, reversible, synthetic Notch, avidity-based, and logic-gated CARs. **F** Manufacturing Challenges: Overcoming limitations using alternative cell sources (iPSC-derived, allogeneic “off-the-shelf” CAR-Ts, and MAIT cells) and in vivo CAR-T generation approaches. These advancements collectively improve CAR-T cell therapy outcomes in solid tumours

Several innovative CAR-T cell engineering strategies have been developed to address antigen heterogeneity and immune escape. Multi-antigen recognition can be achieved through advanced CAR constructs, including bicistronic CARs, tandem CARs (tanCARs), and loopCARs, each employing different structural modifications to improve antigen binding and enhance T-cell activation. Bicistronic CARs introduce two distinct CAR constructs into a single T cell, allowing independent recognition of two antigens and broadening tumour recognition [132, 133]. The triCARs and quad-CARs have been developed to target three or more TAAs, extending the scope of tumour cell recognition and minimising immune evasion [134]. TanCARs, in contrast, fuse two antigen-binding domains in tandem within a single CAR structure, enabling simultaneous engagement with multiple antigens and triggering a more robust immune response (Figs. 3A and 4C) [135]. These approaches are particularly beneficial in tumours with known heterogeneity, such as glioblastoma and lung cancer, where distinct tumour regions may express different antigen profiles [136, 137]. Studies have highlighted the benefits of these multi-targeting approaches. In a study by Puliafito et al., CART19/20, a bispecific CAR-T therapy targeting CD19 and CD20, was engineered to improve CAR-T persistence and mitigate antigen escape. In a phase 1 trial for relapsed/refractory non-Hodgkin lymphoma (R/R NHL), CART19/20 demonstrated a favourable safety profile with no severe CRS or neurotoxicity while achieving an objective response rate (ORR) of 91% and complete remission in 73% of patients [132].

LoopCARs represent an innovative advancement by optimising the spatial organisation of antigen-binding domains. Unlike traditional CAR constructs, which arrange their antigen-binding sites linearly, LoopCARs utilise a flexible linker to connect two antigen-binding domains in a circular configuration, optimising spatial orientation for antigen engagement and enhancing signal transduction efficiency. This structural design improves synapse formation and strengthens T-cell activation, particularly in cases where antigens are variably expressed [138]. Preclinical studies have shown that loopCARs exhibited the highest efficacy among four bispecific CAR-T variants tested, eradicating lymphoma cell lines and significantly prolonging survival in xenograft models [139].

The OR logic gate in CAR-T cell design represents another promising approach. Instead of relying on a single antigen, the OR logic gate in CAR-T cells is designed to recognise two or more tumour antigens simultaneously, increasing the likelihood of effective tumour clearance and mitigating the risk of antigen escape [140, 141]. Next-generation CAR-T cell therapies offer a more adaptable and durable immunotherapy approach by integrating strategies, including multi-targeting CARs and OR logic gate CARs. These innovations not only enhance tumour recognition but also reduce the risk of antigen escape (Fig. 3).

#### Prioritising high-value CAR antigen targets

The efficacy of CAR-based therapies in solid tumours remains limited by TAA heterogeneity and the scarcity of TSAs. Nevertheless, several TAAs exhibit favourable expression profiles and functional roles in tumourigenesis, rendering them crucial targets. Ideal CAR targets for solid tumours should exhibit high and homogeneous expression in malignant tissues, minimal expression in vital organs, and biological roles that confer tumourigenic advantages [142].

HER2 (ERBB2) is one of the most extensively studied TAAs. It encodes a receptor tyrosine kinase that activates PI3K/AKT and MAPK signalling pathways, promoting tumour proliferation, survival, and metastasis [143]. Although HER2 is also expressed at low levels in healthy tissues, it is frequently overexpressed in breast, gastric, and ovarian cancers. In a landmark study by Ahmed et al. (2015), potent anti-tumour activity of HER2-specific CAR-T cells against autologous HER2-positive glioblastoma multiforme (GBM) was shown, including regression of intracranial GBM xenografts in murine models [27]. Nevertheless, its clinical translation has been hindered by on-target/off-tumour toxicity, as highlighted by a fatal case due to low-level HER2 expression in lung epithelium [27].

Glypican-3 (GPC3) is a cell-surface heparan sulfate proteoglycan anchored via a glycosylphosphatidylinositol (GPI) moiety. It is selectively overexpressed in hepatocellular carcinoma (HCC) and certain paediatric embryonal tumours, including yolk sac tumours, while being virtually undetectable in normal adult tissues [144]. It regulates Wnt/β-catenin signalling and supports oncogenesis [144, 145]. GPC3-targeting CAR-T cells with dual cytokine expression of IL-15 and IL-21 exhibited enhanced antitumour activity against HCC, demonstrating superior proliferation, persistence, and tumour regression in GPC3-positive xenograft models [146]. A phase I clinical trial (NCT02905188) is evaluating the feasibility of GPC3-specific CAR-T cells (GLYCAR T cells) in patients with advanced HCC, aiming to determine the maximum tolerated dose, persistence of infused cells, safety profile, and preliminary antitumour efficacy.

Mesothelin (MSLN), overexpressed in mesothelioma, pancreatic adenocarcinoma, and ovarian cancer, is another attractive target. As a glycosylphosphatidylinositol (GPI)-anchored membrane protein, MSLN facilitates metastasis via MUC16 interaction [147]. Phase I studies using MSLN-targeting CAR-T cells have shown tumour localisation and transient tumour stabilisation with an acceptable safety profile [148, 149].

Claudin-18.2, an isoform of claudin proteins, is a tight junction protein isoform predominantly expressed in gastric mucosa and aberrantly upregulated in several gastrointestinal malignancies, including gastric, pancreatic, and oesophageal cancers [150]. Satricabtagene autoleucel (satri-cel; CT041), an autologous CLDN18.2-specific CAR-T cell therapy, was evaluated in the phase I CT041-CG4006 trial (NCT03874897) involving 98 patients with advanced CLDN18.2-positive gastrointestinal cancers. This therapy showed an overall response rate of 38.8% and a disease control rate of 91.8%, with a median progression-free survival of 4.4 months and a median overall survival of 8.8 months. It also exhibited a manageable safety profile, with no dose-limiting toxicities, treatment-related deaths, or neurotoxicity, and cytokine release syndrome in 96.9% of patients, all grade 1–2 [151].

CD70, the ligand of CD27, is another highly promising target, especially in clear cell renal cell carcinoma (ccRCC), glioblastoma, and certain hematologic malignancies. CD70 expression is largely restricted to activated lymphocytes and is aberrantly upregulated in ccRCC [152]. Preclinical studies have demonstrated the potent cytotoxicity of CD70-targeting CAR-T cells in xenograft models of RCC [153]. Notably, CTX130, an allogeneic CD70-targeted CAR-T cell therapy, demonstrated promising preclinical activity and early clinical efficacy in a phase I trial involving 16 patients with relapsed/refractory clear cell renal cell carcinoma (ccRCC). The disease control was achieved in 81.3% of patients, with one patient maintaining a complete response for over 3 years. No dose-limiting toxicities were observed. A next-generation product, CTX131, exhibited enhanced expansion and antitumour activity in preclinical models, further supporting the therapeutic potential of CD70-targeted CAR-T cells in ccRCC and other CD70-positive solid tumours [154].

EGFR and its mutant isoform EGFRvIII are key drivers in glioblastoma. While wild-type EGFR is broadly expressed in normal tissues, EGFRvIII is a tumour-specific deletion variant found exclusively in glioblastoma and some other tumours [155]. A phase I trial reported the feasibility and safety of a single intravenous dose of autologous EGFRvIII-targeted CAR T cells in 10 recurrent glioblastoma patients. One patient achieved stable disease for over 18 months, with peripheral CAR persistence and evidence of tumour trafficking and antigen loss [156]. A detailed case of a 59-year-old patient (NCT02209376) was published, demonstrating 36-month survival post-infusion (far exceeding typical prognosis), with persistent CAR-EGFRvIII cells detectable up to 29 months [157].

Carcinoembryonic antigen (CEA) is a glycoprotein overexpressed in several epithelial cancers, notably colorectal, pancreatic, gastric, and non-small cell lung cancers, with limited expression in normal adult tissues, primarily on the luminal side of the gastrointestinal tract [158]. CEA-targeted CAR-T therapy has shown a favourable safety profile and preliminary efficacy in metastatic colorectal cancer. In a dose-escalation study of 10 patients, no severe adverse events occurred, and most experienced disease stabilisation or tumour shrinkage, with CAR-T cell persistence observed [159]. A phase I trial comparing intraperitoneal and intravenous delivery in 40 solid tumour patients reported manageable toxicity and higher response rates with intraperitoneal infusion, particularly at higher doses, including sustained tumour remission in responders [160]. In the end, several TAAs have emerged as leading candidates for CAR-based immunotherapy in solid tumours, demonstrating strong preclinical rationale and early clinical validation. Optimal target selection, antigen density profiling, and innovative CAR design will be essential to enhance efficacy while minimising toxicity. Table 3 lists the key antigens discussed above, along with additional TAAS targeted in CAR-T cell therapies.

**Table 3:** Key target antigens of CAR-T cell therapy in solid tumours

Antigen	Cancer Expression	Preclinical + Clinical Outcomes	References
HER2 (ERBB2)	Breast, gastric, ovarian, and glioblastoma	Robust GBM xenograft regression; clinical fatal lung toxicity observed	[27, 143]
GPC3	HCC, yolk sac, paediatric tumours	IL-15/IL-21 co-expressing CAR-T cells mediate tumour regression; GLYCAR-T phase I (NCT02905188) ongoing	[144–146]
Mesothelin (MSLN)	Mesothelioma, pancreatic, ovarian	Phase I trials reveal tumour localisation and stabilisation with acceptable safety	[147–149]
Claudin-18.2	Gastric, pancreatic, oesophageal	Satri-cel (CT041) achieved ORR 38.8%, DCR 91.8%, PFS 4.4 mo, OS 8.8 mo; low-grade CRS	[150, 151]
CD70	ccRCC, glioblastoma, hematologic	CTX130 showed 81.3% DCR (1 CR > 3 yrs); CTX131 displayed enhanced expansion and efficacy	[152, 153]
EGFRvIII	Glioblastoma	Clinical trial showed safety, tumour trafficking, one patient stable > 18 mo; notable 36-month survival case	[155–157]
tMUC1	Breast, pancreatic, lung	Preclinical tumour regression in models; clinical partial responses and stable disease in H-score high patients	[161, 162]
CEA	Colorectal, pancreatic, gastric, NSCLC	Phase I showed safety, disease stabilisation, and CAR persistence; i.p. dosing improved ORR and durability	[158–160]
B7-H3 (CD276)	Neuroblastoma, glioblastoma, NSCLC, prostate, breast	GBM xenograft eradication; paediatric phase I safety, tumour-localisation, partial response with CAR expansion; multiple ongoing GBM trials	[163–166]
FAP (Fibroblast Activation Protein-α)	CAF-rich stroma in diverse tumours	Robust stromal depletion in xenografts, low off-tumour activity; crucial to stromal barrier disruption in preclinical models	[167]
ROR1 (Receptor Tyrosine Kinase-like Orphan Receptor 1)	Various solid tumours (lung, breast, ovarian)	A phase I study: Safe with limited toxicity; strong responses in CLL (2/3 patients), minimal activity in solid tumours (1/18 PR); limited tumour infiltration and CAR immunogenicity noted as barriers	[168]
PSMA (Prostate-Specific Membrane Antigen)	Prostate cancer	Phase I trial (NCT03089203) of TGFβ-resistant PSMA-CAR-T cells showed feasibility and preliminary efficacy (≥ 30% PSA decline in 4/13 patients; > 98% decline in one), with manageable CRS and evidence of tumour trafficking; one patient died from grade 4 CRS with sepsis	[169]
GD2 (Ganglioside-2)	Neuroblastoma, diffuse midline glioma	Preclinical intracerebroventricular GD2-CAR activity; FDA Regenerative Medicine designation, ongoing clinical trials	[31]
FRα (Folate Receptor α)	ovarian carcinoma, Breast, lung, and endometrial cancers; limited expression in normal tissues	Second-generation FRα-CARs showed enhanced tumour control and CAR-T survival in ovarian cancer models In triple-negative breast cancer (TNBC), FRα-CAR-T cells showed strong in vitro and in vivo suppression of xenografts	[170, 171]
NKG2D (Natural Killer Group 2, Member D) ligands	Induced in many tumour types under stress	NKG2D/Dap10-12 CAR-T cells demonstrated superior efficacy over clinical analogues in xenograft models, with durable tumour control, long-term survival, and resistance to tumour re-challenge	[172]

### Cell exhaustion and persistence issues

A critical limitation in the success of CAR-T cell therapy for solid tumours is T-cell exhaustion, a state of functional decline that occurs due to prolonged antigen exposure and the suppressive effects of the TME (Fig. 2) [173]. Unlike in haematological malignancies, where CAR-T cells often exhibit robust expansion and persistence, solid tumours subject CAR-T cells to chronic antigen stimulation, leading to premature exhaustion and reduced therapeutic efficacy [174]. Immune checkpoint ligands such as PD-L1, CTLA-4, TIM-3, and LAG-3 expressed by tumour and immunosuppressive cells engage with their respective receptors on CAR-T cells, suppressing activation, reducing cytokine production, promoting dysfunction, and ultimately limiting persistence and anti-tumour efficacy (Fig. 2B) [175–177]. Metabolic constraints (described in sub-Sect. 4.1), including nutrient deprivation and hypoxia, further exacerbate exhaustion by impairing mitochondrial function and driving T cells toward a dysfunctional state (Fig. 2A, F) [178].

Moreover, the efficacy of CAR-T cells in solid tumours is significantly influenced by the selection of T-cell subsets. A major intrinsic challenge is the functional heterogeneity among T-cell subsets, which affects persistence, proliferation, and cytotoxic potential [179]. While CD8 + T cells exhibit strong direct tumour-killing activity, they often suffer from exhaustion and reduced persistence in the hostile TME [180]. In contrast, CD4 + T cells contribute to long-term persistence and cytokine secretion but may not be as effective in direct tumour lysis [181]​. The balance between these subsets is critical, as an improper ratio can lead to suboptimal responses. Additionally, naïve T cells (TN) and Stem cell memory T cells (Tscm) demonstrate superior persistence and self-renewal capabilities compared to effector memory T cells (TEM), which are more cytotoxic but short-lived [182]​. However, selecting subsets with strong persistence often leads to delayed tumour clearance, whereas prioritising highly cytotoxic subsets results in rapid exhaustion and dysfunction.

Another major challenge is the presence of Tregs, which suppress anti-tumour immune responses and limit CAR-T cell activity​ [183]. These intrinsic limitations necessitate optimised subset selection to enhance the balance between persistence, cytotoxicity, and resistance to TME-induced dysfunction.

#### Improving CAR-T cell persistence and resistance to exhaustion

Several engineering strategies have been developed to enhance CAR-T cell persistence and mitigate exhaustion (Fig. 4D). One of the most effective approaches is the incorporation of costimulatory domains, such as 4-1BB (CD137) or CD28, into CAR constructs (second-generation CARs and after). These costimulatory signals promote T cell survival, proliferation, and metabolic fitness, allowing CAR-T cells to sustain their cytotoxic activity over an extended period [184]. 4-1BB-based CAR-T cells have demonstrated enhanced persistence compared to CD28-based CARs, as 4-1BB signalling supports mitochondrial biogenesis and reduces activation-induced cell death [185]. However, CD28-based CARs provide stronger initial activation, making them advantageous in specific therapeutic contexts [186]. Recent efforts have focused on optimising hybrid costimulatory domains that combine the benefits of both signalling pathways to balance rapid activation and long-term persistence (Fig. 1B) [187].

Beyond costimulatory enhancements, genome editing technologies such as CRISPR-Cas9 have been employed to knock out exhaustion-associated genes, thereby improving T-cell functionality [188]. Disrupting the PD-1, LAG-3, or TIM-3 pathways in CAR-T cells prevents inhibitory signalling, restoring their ability to sustain effector functions within the TME [189–191]. Preclinical studies have shown that PD-1 knockout CAR-T cells exhibit superior anti-tumour activity in solid malignancies by resisting checkpoint-mediated suppression [192]. Additionally, using dominant-negative PD-1 receptors or PD-1/CD28 chimeric switch receptors has been explored as an alternative approach, where PD-1 signalling is converted into an activation signal rather than an inhibitory one [193]. These modifications allow CAR-T cells to remain functional despite persistent tumour-derived immunosuppression.

T-cell exhaustion is closely linked to diminished anti-tumour efficacy, as exhausted T cells exhibit reduced proliferative capacity, impaired cytokine production, and defective cytotoxicity [194]. Cytokines are crucial in driving T-cell exhaustion, negatively impacting their anti-tumour response [195]. To counteract this (As mentioned in Sect. 4.1), CAR-T cells have been engineered to secrete specific cytokines. For instance, co-expressing IL-15 or IL-21 in CAR-T cells has been explored to enhance persistence and mitigate exhaustion, thereby improving their anti-tumour efficacy [146]. Similarly, CAR-T cells with dominant-negative TGF-β receptors (dnTGF-βR) resist TGF-β-mediated suppression, allowing them to sustain functionality within the TME [196]. Furthermore, FOXO1 overexpression or TET2 knockout can further enhance CAR-T durability [197, 198].

Optimising CAR-T cell subsets is crucial to maintain the balance between cytotoxicity, persistence, and resistance to exhaustion [199]. A well-defined ratio of CD4 + and CD8 + T cells, such as a 1:1 ratio, has been shown to enhance efficacy by combining the direct tumour-killing ability of CD8 + T cells with the cytokine support provided by CD4 + T cells, particularly Th1-skewed cells that secrete IFN-γ, IL-2, and TNF-α [200, 201]. To further improve cytotoxicity while mitigating exhaustion, selecting CD8 + CD161 + T cells, which express higher levels of granzyme B and perforin, enhances tumour clearance [202].

Beyond subset composition, alternative cell sources offer additional benefits. Prioritising TNs and Tscm-T cells over TEMs and terminally differentiated effector T cells (TEFF) is critical for enhancing CAR-T cell persistence and anti-tumour activity [203, 204]. Tscm cells with high CD62L and CCR7 expression possess superior self-renewal, multipotency, and persistence, making them ideal for CAR-T cell therapies. Compared to conventional CAR-T cells, Tscm-derived CAR-T cells exhibit enhanced expansion, reduced exhaustion, and durable anti-tumour responses due to their oxidative metabolism [205–207]. Altogether, advances in costimulatory domain design, gene editing, cytokine engineering, T-cell subset optimisation, and alternative cell sources offer promising solutions to enhance durability, resistance to immunosuppression, and long-term efficacy in next-generation CAR-T therapies.

### Toxicity and off-target effects

Despite the advancements of CAR T-cell therapy, toxicity and off-target effects remain significant challenges that limit its broader clinical application. Some tumours frequently express antigens that are also found in normal tissues. This antigen overlap increases the risk of "on-target, off-tumour" toxicity, where CAR-T cells attack healthy cells expressing the target antigen, leading to severe adverse effects [208]. For instance, HER2-targeted CAR-T cells have been reported to cause life-threatening pulmonary toxicity due to HER2 expression on lung epithelial cells [209]. Similarly, B7-H3 and mesothelin, promising targets in solid tumours, are also found in normal tissues, though at low levels, making precise targeting essential [210, 211].

In addition to off-target effects, CAR-T therapy can trigger systemic toxicities, such as CRS and ICANS. CRS results from excessive activation of CAR-T cells, leading to uncontrolled cytokine production (e.g., IL-6, IFN-γ, and TNF-α), which can cause fever, hypotension, vascular leakage, and multi-organ dysfunction [212]. Neurotoxicity (ICANS), though less well understood, is believed to be related to blood–brain barrier disruption and cytokine-mediated inflammation, leading to symptoms ranging from confusion and seizures to cerebral oedema and fatal complications [213]. While commonly associated with haematological CAR-T therapies, these toxicities are also a major concern in solid tumours due to prolonged CAR-T activation in the suppressive TME [21].

#### Engineering CAR-T cells for enhanced specificity and safety

Several strategies have been developed to enhance safety and specificity while minimising toxicity. One key approach involves suicide gene switches or small-molecule-controlled "on/off" switches, which allow for external regulation of CAR-T cell activity (Figs. 3B and 4E). Suicide genes, such as inducible caspase-9 (iCasp9), can be triggered by administering a specific small molecule, leading to rapid CAR-T cell apoptosis in cases of severe toxicity [214, 215]. Similarly, small-molecule-controlled CARs enable the temporary activation or deactivation of CAR-T cells based on patient response, reducing systemic toxicities [216].

Reversed (Rev) CARs introduce a novel two-component modular system that separates antigen recognition from activation, relying on bispecific antibody-based target modules (RevTMs). Unlike conventional CARs, RevCAR-T cells remain inactive unless a RevTM bridges the CAR and tumour antigen, providing an ON/OFF control switch. This reduces antigen-independent tonic signalling, limits systemic toxicity, and allows flexible dosing strategies [217].

Other CAR-T platforms focus on precision antigen recognition to enhance specificity and mitigate off-target effects. One such approach is NOT logic gate CARs (iCARs), which integrate inhibitory signalling domains derived from immune checkpoint receptors such as PD-1 or CTLA-4. These receptors suppress CAR-T cell activation upon recognising an off-target antigen, thereby preventing unintended cytotoxicity [218, 219].

Similarly, "IF-Better" logic gate CARs introduce a dynamic activation threshold, requiring high antigen density and a secondary costimulatory signal for full CAR-T activation. Unlike conventional CARs that react to any detectable antigen level, this system ensures preferential targeting of cancer cells while sparing normal cells with low antigen expression [220].

Avidity (Avid) CARs incorporate low-affinity, dual antigen-binding domains that require bivalent interactions to activate, preventing responses to single-antigen-expressing normal cells. This system improves tumour selectivity and has been particularly advantageous in solid tumours, where heterogeneous antigen expression poses a major challenge [221].

AND-gate CARs, also called split-recognition CARs, further enhance tumour specificity by requiring two distinct tumour antigens for full activation. This is achieved by separating the CD3ζ activation and costimulatory domains (e.g., CD28 or 4-1BB) across two distinct CAR constructs. This ensures that activation occurs only when both antigens are co-expressed on the tumour cell. This strategy greatly reduces the risk of off-target toxicity [222, 223]. For example, CAR-T cells engineered to express anti-mesothelin (CD3ζ) and anti-folate receptor alpha (CD28) only exhibit full effector function when both markers are present, preventing reactivity to normal tissues that express only one of the antigens [224].

Beyond these antigen-specific designs, synthetic Notch (synNotch) receptors provide an orthogonal mechanism for tumour targeting by requiring sequential antigen recognition. When the first antigen is encountered, synNotch receptors trigger the expression of a secondary CAR targeting a different antigen, ensuring context-dependent CAR activation [225, 226]. This layered recognition system enhances selectivity, prevents premature T-cell exhaustion, and reduces antigen escape risks [226].

Universal or switchable CAR constructs allow CAR-T cells to adjust their antigen specificity dynamically based on the tumour’s evolving antigen profile [227]. SUPRA-CARs (split, universal, and programmable CARs) exemplify this strategy. They utilise a universal receptor on CAR-T cells that binds to an adapter molecule, directing them to a specific antigen. By modifying the adapter, the targeting specificity of CAR-T cells can be altered in real-time, providing flexibility in addressing tumour antigen heterogeneity and minimising immune evasion [228]. The next generation of CAR constructs represents a significant advancement in engineered T-cell therapies, integrating enhanced specificity, safety mechanisms, and multi-antigen targeting capabilities. As illustrated in Fig. 3.

Localised CAR-T delivery methods, which restrict CAR-T cells to the tumour site, reduce systemic exposure. Intratumoral injections or regional administration (e.g., intrapleural, intraperitoneal, or intravesical delivery) ensure that CAR-T cells remain concentrated within the TME, limiting off-target effects. This strategy has been particularly beneficial in treating tumours in confined anatomical spaces, such as glioblastomas (intracranial injections) or mesotheliomas (intrapleural injections), where the systemic circulation of CAR-T cells would otherwise increase toxicity risks [229]. Collectively, these advancements in CAR-T engineering, through logic gating, modular activation, affinity optimisation, and localised delivery of CAR-T cells, offer highly refined tumour targeting strategies, enhanced safety profiles, and greater therapeutic control.

### Manufacturing and cost challenges

One of the major barriers to the widespread implementation of CAR-T cell therapy for solid tumours is the complexity and high manufacturing cost. Traditional autologous CAR-T cell therapy involves extracting T cells from each patient, genetically engineering them to express a CAR, expanding them ex vivo, and then reinfusing them into the patient [230]. This personalised approach is time-consuming and labour-intensive and requires specialised infrastructure, stringent quality control measures, and extensive clinical monitoring [231]. Consequently, CAR-T cell therapy remains prohibitively expensive, with treatment costs exceeding $350,000–$500,000 per patient in some cases [232]. Moreover, because production takes several weeks, patients with aggressive tumours may not survive long enough to receive their engineered CAR-T cells, highlighting the urgent need for faster and more cost-effective manufacturing solutions [233].

#### Advancing scalable and cost-effective CAR-T manufacturing platforms

In response to manufacturing limitations, researchers are advancing allogeneic (off-the-shelf) CAR-T cell therapies, which leverage T cells from healthy donors as a scalable alternative to autologous approaches (Fig. 4F). This approach significantly reduces production time and cost by enabling mass manufacturing of universal CAR-T cells that can be stored and administered as needed [234]. However, a major obstacle to allogeneic CAR-T therapy is graft-versus-host disease (GVHD), where donor T cells recognise the patient’s tissues as foreign and initiate an immune attack [235]. To overcome this, gene-editing technologies such as CRISPR-Cas9, TALEN, and zinc-finger nucleases (ZFNs) are being used to eliminate endogenous TCRs, preventing GVHD and allowing for safe allogeneic CAR-T administration [236]. For instance, MHC knock out (KO) can reduce the risk of immune rejection [237]. Deleting Fas or B2M genes in the allogenic CAR-T cells demonstrated improved survival. Specifically, Fas-deleted CAR-T cells showed resistance to NK cell rejection, while B2M-deleted CAR-T cells were vulnerable to rejection by NK cells. However, CD3–Fas–CAR-T cells, which lacked both Fas and B2M, outperformed other modifications in controlling leukaemia growth in mice, even under immune rejection by both NK cells and T cells, underscoring an innovative strategy for addressing the challenges of GVHD in CAR-T cell therapy [238].

In vivo CAR-T cell generation strategies bypass the need for ex vivo cell manipulation. The process typically employs viral or non-viral delivery systems, such as lentiviral vectors, adeno-associated viruses (AAV), and polymer- or lipid-based nanoparticles, to introduce CAR transgenes into T cells, enabling them to express CARs inside the body​ [239, 240]. Preclinical studies have demonstrated the feasibility of in vivo-generated CAR-T cells, with significant tumour regression observed in murine models following AAV-mediated delivery of CAR transgenes​ [241]. Additionally, lentiviral platforms have been optimised to selectively transduce CD4 + and CD8 + T cells, improving transgene specificity while reducing off-target effects [242]​. A recent study utilising a lentiviral-based system, VivoVec, successfully engineered CAR-T cells in vivo, achieving expansion and persistence without preconditioning regimens [243]​. Despite these advancements, clinical translation remains in its early stages and presents potential safety concerns, including insertional mutagenesis from integrating viral vectors and the risk of modifying non-T cells [244]. To mitigate these risks, researchers are exploring non-integrating delivery systems, such as lipid nanoparticles carrying CAR-encoding mRNA, which offer transient CAR expression and enhanced safety [245]​. Furthermore, incorporating gene-editing tools like CRISPR/Cas9 could refine in vivo CAR-T strategies, allowing precise genomic integration and reducing the likelihood of adverse events​ [246].

T-cells can be derived from alternative T-cell sources. Induced pluripotent stem cells (iPSCs) offer a scalable solution to the challenges of autologous CAR-T cell production, which relies on harvesting T-cells from individual patients. By engineering iPSCs to express CARs, a continuous supply of CAR-T cells is provided from a single iPSC clone, ensuring uniformity and reducing manufacturing complexities. This approach enables the creation of"off-the-shelf"CAR-T therapies, potentially lowering costs and accelerating treatment availability [247]. In a study, Wang et al. utilized a 3D-organoid culture system to differentiate human CAR + iPSCs into functional CAR-T cells. These cells exhibited potent anti-tumour activity in vivo, prolonging the survival of mice with CD19 + human tumour xenografts [248].

T-cells can also be derived from Mucosal-associated invariant T (MAIT). Derived from the human placenta, MAIT cells are abundant and can be expanded using advanced 3D cell expansion technologies, facilitating scalable production of off-the-shelf therapeutic products [249]. This approach reduces the high production costs and complexities associated with autologous CAR-T cell therapies and ensures batch-to-batch consistency, enhancing product reliability. Furthermore, MAIT cells exhibit potent effector functions and express high levels of various chemokine receptors, enabling efficient migration to tumour sites. Notably, their unique properties minimise the risk of GVHD, a significant concern in allogeneic settings [250].

Optimising bioreactor systems and automated cell culture platforms allows for large-scale, standardised CAR-T production with minimal human intervention [251]. Advances in cryopreservation techniques also enable long-term storage and transportation of CAR-T products, increasing accessibility to patients worldwide [252]. Overall, manufacturing and cost remain major hurdles for CAR-T therapy. However, advances in allogeneic CAR-T cells, in vivo generation, alternative cell sources, and automation promise greater affordability and scalability. These innovations could transform CAR-T therapy into a widely accessible, cost-effective cancer treatment. Figure 4 illustrates potential strategies to overcome CAR-T cell challenges in solid tumours.

## Beyond CAR-T: emerging alternative cellular therapies

### CAR-natural killer cells

The limitations of CAR-T cells in solid tumours have necessitated the development of alternative cell-based therapies. NK cells play a pivotal role in innate immunity, targeting virus-infected and malignant cells through stress-induced ligands (e.g., NKG2D ligands: MICA, MICB, ULBPs) or via the'missing-self'mechanism, which identifies the absence of HLA class I molecules, a common tumour-immune evasion strategy​ [253]. Unlike CAR-T cells, which require specific antigen expression, CAR-NK cells exhibit intrinsic cytotoxicity and can eliminate tumour cells irrespective of target antigen presence. NK cells execute cytotoxicity through multiple pathways: direct lysis via perforin and granzyme release, death receptor-mediated apoptosis (FasL, TRAIL), and antibody-dependent cellular cytotoxicity (ADCC) through CD16​ [254]. CAR-NK cells also leverage natural cytotoxicity receptors (NCRs: NKp46, NKp44, NKp30), co-stimulatory receptors (DNAM-1), and activating killer cell immunoglobulin-like receptors (KIRs: KIR2DS1, KIR2DS4, KIR2DL4), enabling broad-spectrum tumour targeting [255]. Additionally, NK cells produce IFN-γ, activating DCs, enhancing IL-12 production, and promoting Th1 polarisation and CTL activation [256, 257]. They also secrete chemokines like CCL5, recruiting monocytes and DCs to the TME [258].

CAR engineering enhances NK cell therapeutic potential by equipping them with CARs adapted from CAR-T cell designs [259]. CAR-NK cells consist of an extracellular antigen-recognition domain (typically an scFv from monoclonal antibodies targeting TAAs or TSAs), a transmembrane domain (e.g., CD28, CD8α, or NKG2D for stability), and an intracellular signalling domain incorporating CD3ζ and NK-specific co-stimulatory motifs (2B4/CD244, DAP10, DAP12) to enhance cytotoxicity and ADCC [260–264]. By combining CAR-dependent and CAR-independent mechanisms, CAR-NK cells offer a potent and versatile approach to tumour eradication. Table 4 summarises and compares key characteristics between CAR-T, CAR-NK, CAR-M, γδ CAR-T, and CAR-NKT Cells in Solid Tumour Therapy.

**Table 4:** Comparative Characteristics of CAR-T, CAR-NK, CAR-M, γδ CAR-T, and CAR-NKT Cells in Solid Tumour Therapy

Aspect	CAR-T Cells	CAR-NK Cells	CAR-Ms	γδ CAR-T Cells	CAR-NKT Cells
Mechanism of Cytotoxicity	Perforin-granzyme-mediated apoptosis, death receptor activation (Fas-FasL), cytokine secretion (IFN-γ, TNF-α) [265]	Perforin-granzyme pathway, death receptor-mediated apoptosis (FasL, TRAIL), ADCC (CD16), NK receptor signalling (NKG2D, NCRs, KIRs), cytokine secretion (IFN-γ) [254]	Phagocytosis (FcRγ, Megf10, MerTK), immune modulation (IL-12, IFN-γ, TNF-α), TME remodelling (ROS, MMPs), direct cytotoxicity (perforins, serine proteases, TRAIL) [266, 267]	CAR-directed and γδ TCR-mediated cytotoxicity; targets both tumour antigens and stress ligands; ADCC via Fc receptors; cytokine secretion (IFN-γ, TNF-α) [268–270]	Dual recognition via CAR and invariant NKT TCR; secretes Th1 cytokines; strong cytotoxicity with lower on-target toxicity risk; modulates innate and adaptive immunity [271–273]
Persistence & Memory	Long-lasting, can form memory cells, leading to durable responses, but potential for prolonged toxicity [25, 36]	Shorter persistence, reducing prolonged toxicity risk but potentially limiting efficacy [36, 274]	Limited persistence, but contributes to sustained immune response via antigen presentation and TME remodelling [267]	Moderate persistence; memory-like phenotype can be induced; expansion and persistence are current optimisation targets [269]	Exhibits memory features with controlled persistence; avoids long-term toxicity; enhanced expansion rates [273, 275, 276]
Safety Profile	High risk of CRS and neurotoxicity; allogeneic CAR-T may cause GVHD [215, 216, 277]	Lower CRS and neurotoxicity risk, and rarely cause GVHD [277]	Low CRS/neurotoxicity risk; immune modulation may reduce TME suppression [278]	Low risk of GVHD due to MHC-independence; well tolerated in early trials (e.g., ADI-001) [279, 280]	Minimal CRS or neurotoxicity observed in early-phase trials; low GVHD risk [271]
Tumour Infiltration	Limited, especially in solid tumours due to poor penetration and antigen heterogeneity [76, 281]	Moderate; depends on cytokine signalling and innate cytotoxicity mechanisms [255, 282]	Superior; actively infiltrates solid tumours and remodels the TME [278]	Enhanced infiltration into solid tumours in preclinical models (e.g., glioblastoma, HCC) [283]	Enhanced infiltration and tumour localisation; effective in solid and haematologic malignancies [275]
Antigen Dependence	Highly antigen-dependent and requires target expression for efficacy [26, 284, 285]	Less antigen-dependent and combines CAR-driven and innate immune mechanisms [253, 255, 282]	Least antigen-dependent; eliminates tumour cells via phagocytosis, avoiding antigen escape [266]	Partially antigen-independent due to stress ligand recognition by γδ TCR; less affected by antigen heterogeneity [268–270]	Dual targeting enables recognition of broader tumour types; reduced risk of immune escape [271, 272, 286]
TME Resistance	Suppressed by immunosuppressive factors in the TME (TGF-β, IL-10) [39, 43, 49]	Moderately affected; functions better than CAR-T in suppressive TMEs [36]	Actively remodels the TME, enhances immune infiltration, and counteracts immune evasion [287]	Modulates TME via IFN-γ, TNF-α; activates DCs, NKs, macrophages; resistant to suppression [270]	Improves immune cell infiltration and TME modulation; mitigates immunosuppression [288]
Manufacturing Process	Mostly autologous, complex, time-consuming, and costly; allogeneic approaches face HLA matching and GVHD risks [231, 234]	More cost-effective;"off-the-shelf"availability using PBMCs, UCB, NK cell lines, or iPSCs due to lower GVHD risk [285, 289]	Challenging due to macrophage differentiation complexity; scalable potential but requires optimisation [290]	Allogeneic γδ T cells from healthy donors are feasible; clinical-grade expansion remains an optimisation area [279, 280]	Universal CAR-NKT cell production via HSCs and gene editing is possible; early trials support scalable manufacturing [271, 272]

#### CAR-natural killer cells in cancer immunotherapy

CAR-NK cell therapy has shown promising potential in haematological malignancies, particularly B-cell malignancies, AML, and MM. In a pivotal phase I/II trial, cord blood-derived anti-CD19 CAR-NK cells induced a 73% overall response rate in R/R B-cell malignancies, including NHL and CLL, with no cases of CRS or neurotoxicity (NCT03056339) [291]. Similarly, a phase I trial of CD33-targeted CAR-NK cells in R/R AML reported MRD-negative remission in 60% of patients without severe toxicity or GVHD (NCT05008575) [292]. Clinical evidence for CAR-NK therapy in solid tumours remains limited. Early-phase trials and preclinical studies indicate promising therapeutic potential. A phase I trial of HER2-targeted NK-92/5.28.z cells in recurrent glioblastoma demonstrated no severe toxicities and induced stable disease in five patients, with CD8⁺ T-cell infiltration correlating with longer progression-free survival [293]. Preclinical models have shown strong anti-tumour responses in various solid malignancies: TF- or PD-L1-targeted CAR-NK cells in TNBC [294], c-Met CAR-NK cells with DAP10 co-stimulation in LUAD [295], and DLL3-specific CAR-NK cells in SCLC, which exhibited effective tumour suppression and immune infiltration [296].

#### Challenges and optimisation strategies in car-natural killer cells therapy

##### Persistence

**Fig. 5:**
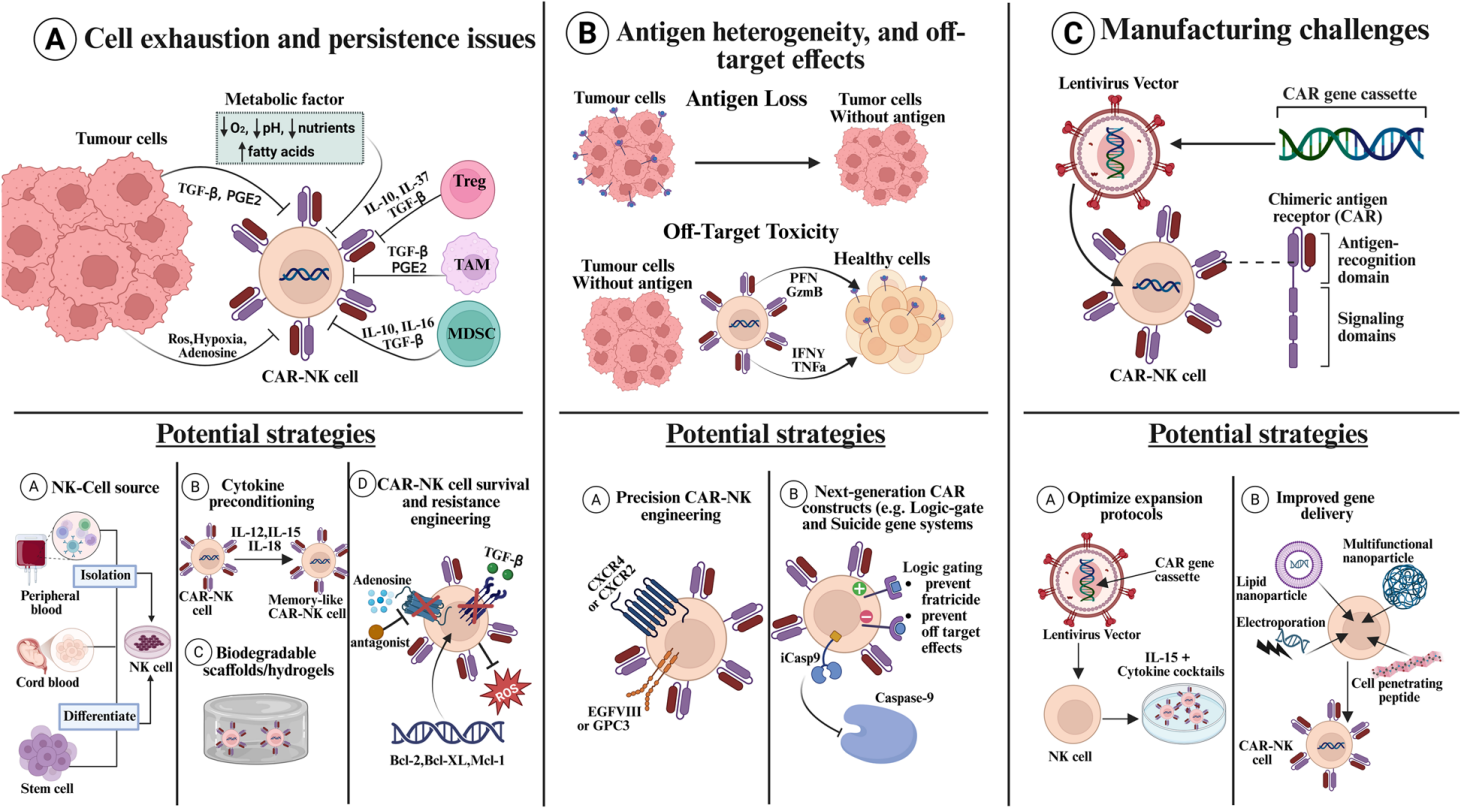
This figure illustrates the major challenges of CAR-NK cell therapy in solid tumours and the strategies designed to address these obstacles. **A** The first challenge is the lack of persistence, where CAR-NK cells exhibit limited durability due to the immunosuppressive TME. Factors such as metabolic stress (hypoxia, low pH, nutrient depletion), ROS, and inhibitory cytokines (TGF-β, PGE2, IL-10, IL-16) contribute to CAR-NK cell exhaustion, while suppressive immune cells, including TAMs, MDSCs, and Tregs, further inhibit CAR-NK activity. Strategies to overcome this limitation include sourcing NK cells from diverse origins such as peripheral blood, umbilical cord blood, or stem cells, cytokine preconditioning (IL-2, IL-12, IL-15, IL-18) to enhance CAR-NK memory-like function, utilising biodegradable scaffolds and hydrogels for in vivo support, and engineering CAR-NK cells for survival and resistance by targeting adenosine and TGF-β signalling or overexpressing pro-survival factors like Bcl-2, Bcl-XL, and Mcl-1. The second challenge is antigen heterogeneity and off-target effects. To mitigate these effects, precision CAR-NK engineering can target specific tumour markers, and logic-gated or suicide gene systems, such as inducible caspase-9 (iCasp9), can be employed to prevent fratricide and minimise off-target toxicity. The third challenge, manufacturing limitations, stems from the inefficiency and cost of genetically modifying NK cells using viral (e.g., lentivirus) or non-viral systems. Strategies to optimise manufacturing include refining expansion protocols with IL-15 and cytokine cocktails to improve CAR-NK proliferation, as well as enhancing gene delivery methods through electroporation, lipid nanoparticles, multifunctional nanoparticles, and cell-penetrating peptides to improve transduction efficiency

Despite their immense promise, CAR-NK cells are not without limitations. One of the most significant challenges is the limited persistence of CAR-NK cells post-administration (Fig. 5A). Unlike CAR-T cells, NK cells lack the long-lasting memory phenotype inherent to adaptive immune cells, compromising their ability to sustain anti-tumour responses over time​​. Furthermore, the immunosuppressive TME poses critical barriers for both cell types, but CAR-NK cells are particularly vulnerable to inhibitory signals [274].

Innovative strategies have been developed to address these limitations. One approach involves engineering CAR-NK cells with cytokine secretion, such as IL-15, to maintain therapeutic efficacy. However, it is often associated with cytokine-mediated systemic toxicity [297, 298]. Other alternatives include the pre-activation of NK cells with cytokine preconditioning (e.g., IL-12, IL-15, and IL-18), which can generate memory-like NK cells with enhanced persistence, metabolic fitness, and prolonged anti-tumour responses [299]. Another approach includes embedding CAR-NK cells in biodegradable scaffolds or hydrogels that locally release cytokines (e.g., IL-2, IL-15, IL-21), supporting in vivo survival while minimising systemic toxicity [300, 301].

Other strategies include genetic engineering that introduces anti-apoptotic genes such as Bcl-2, Bcl-xL, or Mcl-1. These can enhance NK cell survival and prolong their persistence in vivo by counteracting apoptosis-inducing signals within the TME [302]. Moreover, TGFβresistant receptors, CAR-NK cells, or A2A adenosine receptor blockade have shown preclinical promise [303]. Moreover, engineering NK cells to resist ROS or express antioxidants can neutralise TME-induced metabolic suppression, further bolstering their anti-tumour activity [304]. Furthermore, the differentiation of CAR-NK cells from allogeneic hematopoietic stem cells (HSCs) or iPSCs has emerged as a promising avenue for producing NK cells with extended longevity and self-renewal capacity, further addressing the challenge of persistence [305].

##### Antigen heterogeneity and loss, and the off-target effects

Tumour heterogeneity and antigen escape pose challenges for all CAR-based therapies, including CAR-NK cells (Fig. 5B). The loss or downregulation of target antigens, such as NKG2D ligands, can lead to therapy resistance [306]​​. Potential strategies include multiplex targeting and combinatorial antigen recognition. Engineering of NK cells to express bispecific or tandem CARs enables the recognition of multiple TAAs, reducing the likelihood of tumour escape due to single-antigen loss [307]. Combinatorial antigen recognition, such as logic-gated approaches (e.g., AND/OR gating systems), enables selective activation of NK cells only when multiple tumour-associated antigens are present, thereby improving specificity and reducing off-target toxicity [308].

Off-target effects in CAR-NK cell therapy present significant challenges, as unintended immune responses can lead to toxicity and damage to healthy tissues. A primary concern is cross-reactivity, where CAR-NK cells target normal cells expressing low levels of TAAs, resulting in on-target, off-tumour toxicity, often demonstrated in CAR-T cell therapies that target antigens like CD19, leading to B-cell aplasia and neurotoxicity due to expression in normal tissues [309]. While CAR-NK cells generally have a safer profile, the potential for off-target effects remains, necessitating careful antigen selection and engineering strategies to enhance specificity [310].

Multiple strategies are explored to mitigate off-target toxicities. Suicide gene systems, such as inducible caspase-9 (iCasp9), can be incorporated into CAR-NK cells to enable the selective depletion of engineered NK cells if unintended toxicities arise [311]. The iCasp9 system functions by leveraging an inducible form of the caspase-9 enzyme, which is a key initiator of the apoptotic (programmed cell death) pathway [312]. Furthermore, advanced CAR designs, including AND-gate or NOT-gate CAR constructs, further improve specificity by requiring dual-antigen recognition or integrating inhibitory signals to avoid attacking normal cells [313].

Careful selection of truly tumour-specific antigens is fundamental to minimising off-target toxicity [314]. For example, glypican3 (GPC3) is overexpressed in hepatocellular carcinoma. At the same time, its expression in normal adult tissues is negligible, which enables engineered immune cells to selectively target malignant liver cells without damaging healthy tissue [315]. Similarly, the mutant form EGFRvIII is uniquely present in glioblastoma, providing a tumour-restricted target that minimises the risk of neurotoxicity by sparing normal brain cells [155]. The proteogenomic approach is key for identifying novel tumour-specific antigens by integrating genomic and proteomic data to pinpoint biomarkers uniquely altered in cancer cells [316].

Beyond enhancing intrinsic cytotoxicity, modifying chemokine receptor expression on NK cells is another strategy to improve their trafficking to tumour sites. For example, transducing NK cells with CXCR4 or CXCR2 has enhanced their migration toward chemokine gradients produced by tumours, thereby improving in vivo tumour infiltration and efficacy [317]. Together, these approaches demonstrate the multifaceted strategies pursued to optimise CAR-NK cell therapies and minimise off-targets.

##### Manufacturing challenges

Manufacturing CAR-NK cell therapies poses some challenges due to variability in NK cell sources, low transduction efficiencies, and difficulties in ex vivo expansion while maintaining functional integrity (Fig. 5C). NK cells are generally more difficult to expand ex vivo than T cells and require specialised protocols and feeder systems to achieve clinically relevant numbers [37]. Primary NK cells, for instance, often exhibit donor-to-donor variability and limited proliferative capacity, complicating scale-up and standardisation. Moreover, although NK92 cells offer an “off-the-shelf” alternative with robust expansion kinetics, they require rigorous irradiation protocols to mitigate potential tumourigenicity, which can impact therapeutic potency [289].

Several innovative therapeutic strategies are being developed to overcome challenges in CAR-NK cell manufacturing. For instance, feeder-cell systems employing genetically modified K562 cells expressing membrane-bound IL-21 and 4-1BB ligand have significantly enhanced NK cell proliferation and activation ex vivo [318]. Optimised cytokine cocktails incorporating IL-15, often combined with IL-12 or IL-21, have been shown to improve NK cell expansion, survival, and cytotoxic function [319]. Additionally, advanced non-viral gene delivery methods such as the Sleeping Beauty transposon system and electroporation-based mRNA transfection offer safer and more efficient alternatives to viral vectors, enabling stable or transient CAR expression with reduced genotoxicity risks [320, 321]. In a recent study, in vivo programming of NK cells was achieved using mRNA-based delivery of CAR constructs fused to natural cytotoxic receptors, including NKp30, NKp44, and NKp46. These CARs effectively engaged endogenous signalling adaptors to activate NK cell-mediated tumour lysis and cytokine production. Notably, stable expression of the NKp44-based CAR was dependent on the presence of the NK-specific adaptor DAP12, enabling selective and potent in situ activation of NK cells while minimising off-target effects in healthy tissues. This work establishes a foundational method for future in vivo CAR-NK cell engineering, providing a cell–type–restricted and programmable platform that could overcome manufacturing challenges and improve therapeutic efficacy in solid tumours [321]. The integration of scalable, GMP-compliant bioreactor platforms with optimised culture conditions has become essential for producing consistent, high-quality CAR-NK cell products at clinical scale. Coupled with robust cryopreservation protocols that preserve cell viability and functionality, these advancements address critical manufacturing bottlenecks and support the standardised production of CAR-NK therapies, thereby facilitating their clinical translation in oncology [322].

CAR-NK therapy rapidly advances with genetic engineering breakthroughs, novel targets, and improved manufacturing. Their versatility, safety, and scalability highlight their potential in next-generation cancer immunotherapy. Ongoing research aims to enhance persistence, overcome the TME, and expand applications to a broader range of tumour types, positioning CAR-NK cells as a potent and versatile approach in solid tumour therapy. Figure 5 illustrates strategies to overcome the challenges of CAR-NK Cell Therapies in Solid Tumours.

### CAR-macrophages

Macrophages play a crucial role in cancer immunity due to their dual function in promoting and inhibiting tumour progression. Within the TME, TAMs exhibit plasticity, shifting between the pro-inflammatory M1 phenotype, which supports tumour eradication through antigen presentation and cytokine secretion, and the immunosuppressive M2 phenotype, which promotes tumour growth, angiogenesis, and immune evasion​ [323].

CAR in CAR-Ms consists of four key domains, each tailored to optimise macrophage-specific anti-tumour functions. The extracellular antigen recognition domain is typically a scFv derived from an antibody. This is followed by a hinge or spacer region, often derived from CD8α, IgG1, or CD28, which provides flexibility and improves antigen binding. The transmembrane domain, usually sourced from CD28, CD8α, or FcγRI (CD64), anchors the receptor within the macrophage membrane. The intracellular signalling domain is distinct from those found in CAR-T and CAR-NK cells, incorporating macrophage-specific elements such as Fc receptor γ-chain (FcRγ) or Megf10 to enhance phagocytosis, CD3ζ for immune activation, and MerTK or Bai1 to promote macrophage-mediated tumour elimination. Some constructs also integrate PI3K signalling motifs to improve macrophage survival within the TME [324].

CAR-Ms eliminate tumours through enhanced phagocytosis, immune modulation, and TME remodelling. Unlike CAR-T or CAR-NK cells, CAR-Ms directly engulf tumour cells via FcRγ, Megf10, or MerTK signalling upon antigen recognition [266] (Table 4). They maintain a pro-inflammatory M1 phenotype, secreting TNF-α, IL-12, and IFN-γ while downregulating immunosuppressive factors like IL-10 and TGF-β. CAR-Ms also disrupt the TME by releasing ROS and matrix metalloproteinases (MMPs) to degrade the extracellular matrix, neutralising immune checkpoints and improving immune infiltration. As antigen-presenting cells, they activate T cells and recruit immune effectors through chemokine secretion. Additionally, they exert direct cytotoxicity via perforins, serine proteases, and TRAIL-mediated apoptosis. This multifaceted approach positions CAR-Ms as a powerful tool for solid tumour immunotherapy, though challenges like persistence and TME resistance remain [324].

CAR-Ms offer a novel immunotherapeutic strategy to overcome the suppressive TME​. In contrast to CAR-T cells, which face limitations in solid tumours due to poor infiltration, antigen heterogeneity, and immune evasion mechanisms, CAR-Ms can penetrate solid tumour masses more efficiently. Unlike T cells, macrophages do not rely on MHC recognition, allowing them to circumvent common resistance mechanisms such as antigen escape​ [267]. Furthermore, while CAR-T cells induce tumour cell lysis via perforin and granzymes, CAR-Ms eliminate tumour cells primarily through phagocytosis and subsequent antigen presentation, potentially leading to a broader and more sustained immune response [281]​. Similarly, while CAR-NK cells offer advantages such as ADCC mediation and a lower risk of GVHD, their limited in vivo persistence and susceptibility to TME-induced suppression remain key challenges​ [325]. Conversely, CAR-Ms can actively modulate the TME by secreting pro-inflammatory cytokines and chemokines, enhancing the recruitment and activation of other immune cells [326].

#### CAR-macrophages in cancer immunotherapy

Preclinical studies have highlighted the therapeutic potential of CAR-M therapy in both haematological and solid tumours. Initially proposed by June and Gill in 2017, CAR-M constructs targeting CD19, mesothelin, and HER2 have demonstrated robust phagocytic and cytotoxic activity against antigen-expressing tumour cells [278, 290, 327]. Notably, anti-HER2 CAR-Ms elicited selective cytotoxicity in breast and ovarian cancer models, while integration of CD147 signalling enhanced MMP expression, facilitating ECM degradation and T-cell infiltration [287, 328].

The translation of CAR-M therapy into clinical settings has been marked by promising early-phase trials, particularly for solid tumours, where CAR-T therapies face significant challenges. The first-in-human clinical trial (NCT04660929), initiated by Carisma Therapeutics, evaluated CT-0508, a HER2-targeting autologous CAR-M product derived from peripheral blood monocytes. Preliminary findings indicated a favourable safety profile, with no dose-limiting toxicities, severe CRS, or ICANS. Among 14 patients with HER2-overexpressing tumours (breast and gastroesophageal cancer), 44% of those with HER2 3 + tumours (4 of 9 patients) achieved stable disease as the best overall response at 8 weeks, while no meaningful activity was observed in the HER2 2 + population. Disease progression correlated with lower HER2 expression levels. The treatment primarily induced infusion reactions and mild grade 2 CRS, both manageable. These findings demonstrate the preliminary safety, tolerability, and manufacturing feasibility of CT-0508 for HER2 + tumours [329, 330]. A second early-phase I trial (NCT06224738), initiated in March 2024, is investigating human HER2-CAR-M therapy in advanced HER2-positive gastric cancer with peritoneal metastases. While patient enrolment is ongoing, this trial aims to assess therapeutic efficacy and safety in a broader patient cohort [331]. CAR-M therapy shows promise in addressing limitations of CAR-T and CAR-NK approaches in solid tumours, yet it faces challenges, such as limited persistence and tumour infiltration, that must be the focus of future research.

#### Challenges and optimisation strategies in CAR-macrophage cell therapy

##### Tumour infiltration

**Fig. 6:**
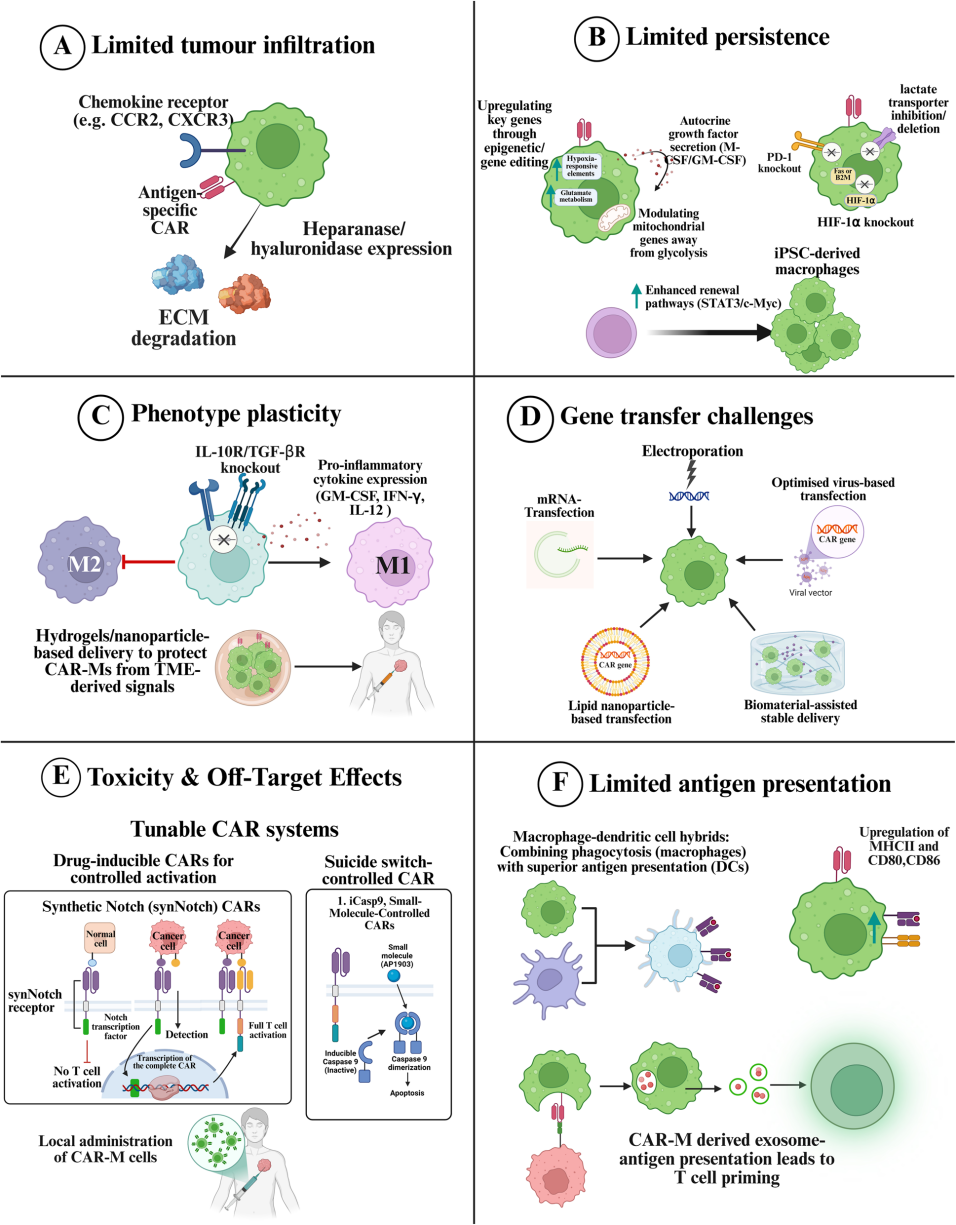
This figure highlights six key challenges in chimeric antigen receptor macrophage (CAR-M) therapy and corresponding strategies to enhance anti-tumour efficacy. **A** Limited Tumour Infiltration: CAR-Ms struggle to penetrate tumours due to the ECM. Engineering chemokine receptors (CCR2, CXCR3) and ECM-degrading enzymes (heparanase, hyaluronidase) improve infiltration. **B** Limited Persistence: CAR-M longevity is enhanced through PD-1 knockout, metabolic reprogramming (HIF-1α knockout, lactate transporter inhibition), and iPSC-derived macrophages with improved renewal pathways (STAT3/c-Myc). **C** Phenotype Plasticity: Preventing M2 polarisation via IL-10R/TGF-βR knockout and reinforcing M1 activation with pro-inflammatory cytokines (GM-CSF, IFN-γ, IL-12) enhances CAR-M functionality. Hydrogel/nanoparticle delivery protects CAR-Ms from immunosuppressive TME signals. **D** Gene Transfer Challenges: Efficient gene delivery is achieved via electroporation-based mRNA transfection, lipid nanoparticles, optimised viral vectors, and biomaterial-assisted stable delivery. **E** Toxicity & Off-Target Effects: Safety is improved with tunable CAR systems, including drug-inducible and suicide switch-controlled CARs (e.g., iCasp9). Local CAR-T administration further reduces systemic toxicity. **F** Limited Antigen Presentation: Enhancing antigen presentation involves generating macrophage-dendritic cell hybrids (upregulating MHC-II, CD80, CD86) and CAR-M-derived exosomes to prime T cells. These innovations aim to optimise CAR-M therapy for improved tumour targeting, persistence, and safety

A major limitation facing CAR-Ms in solid tumours stems from the TME​​ [332]. Potential strategies to enhance CAR-Ms'efficacy in solid tumours include several innovative approaches. One approach involves engineering CAR-Ms to express matrix-remodelling enzymes, such as hyaluronidase or heparanase, which facilitate the degradation of ECM components, thereby improving CAR-M infiltration into tumour tissue (Fig. 6A) [333].

Another approach is enhancing macrophage homing to tumours by upregulating chemokine receptors like CCR2 or CXCR3, which can significantly improve CAR-M migration and retention at tumour sites, thereby boosting therapeutic efficacy. These strategies collectively aim to optimise CAR-M therapy and overcome the challenges of treating solid tumours [334].

##### Limited persistence

Another challenge is the limited persistence and expansion of CAR-Ms in vivo, as macrophages lack the proliferative capabilities of T cells, necessitating repeated administrations for sustained therapeutic effects​ [335]. Several strategies can address limited persistence and exhaustion. For instance, deleting PD-1 or CTLA-4 can prevent engagement with corresponding ligands expressed on tumour cells, thereby inhibiting cell exhaustion [336].

Metabolic reprogramming techniques, such as targeting lactic acid accumulation, can counteract immunosuppressive barriers. To overcome these challenges of metabolic dysregulation of the TME, strategies such as inhibiting lactate transporters (e.g., monocarboxylate transporters MCT1 and MCT4) or lactate dehydrogenase (LDH) can reduce lactate accumulation and restore macrophage immunostimulatory functions [337]. Another promising approach involves reprogramming CAR-Macrophages to sustain glutamine metabolism by upregulating glutaminolysis, enhancing α-KG production, and promoting an M1-like phenotype, ensuring sustained activation and tumouricidal function in the nutrient-deprived TME [338]. Furthermore, other strategies focus on increasing CAR-M resistance to hypoxia by overexpressing hypoxia-responsive elements or incorporating oxygen-carrying nanoparticles, which enhance cell survival and function in the oxygen-deprived TME [339, 340]. Moreover, enhancing oxidative phosphorylation (OXPHOS) in CAR-Ms by overexpressing key mitochondrial enzymes can shift their metabolic state away from glycolysis, thereby improving their persistence and anti-tumour activity [341].

Genetic modifications such as overexpressing anti-apoptotic molecules like Bcl-2 or Bcl-xL, inhibit programmed cell death, and promot sustained survival of macrophage [342]. Additionally, engineering CAR-Ms to secrete autocrine growth factors, such as macrophage colony-stimulating factor (M-CSF) or granulocyte–macrophage colony-stimulating factor (GM-CSF), can enhance their in vivo expansion and maintenance [343].

Finally, using iPSC-derived macrophages provides a renewable source of CAR-Ms with improved persistence and scalability. These iPSC-derived macrophages can be engineered to express self-renewal signalling pathways, such as STAT3 or c-Myc, which promote their longevity and functional stability in the TME [344, 345]. Additionally, periodic reinfusion of CAR-Ms derived from precursor cells, such as monocytes or hematopoietic stem cells (HSCs), can sustain therapeutic efficacy by continuously replenishing the macrophage population (Fig. 6B) [346].

##### Phenotype plasticity

The plasticity of TAMs poses a significant challenge in CAR-M therapy, as engineered macrophages that initially exhibit an M1-like pro-inflammatory phenotype can be reprogrammed into an immunosuppressive M2-like state due to exposure to TME-derived cytokines such as IL-10 and TGF-β [347]. Several strategies have been proposed to counteract this phenotypic drift and maintain CAR-Ms in an anti-tumourigenic state (Fig. 6C). One approach involves genetically modifying CAR-Ms to resist TME-mediated polarisation by knocking out key immunosuppressive signalling pathways, such as the IL-10 receptor (IL-10R) or TGF-β receptor (TGFBR), preventing these cytokines from inducing an M2-like phenotype [196]. Alternatively, CAR-Ms can be engineered to constitutively express M1-polarising cytokines, such as GM-CSF, IFN-γ, and IL-12, which can reinforce their pro-inflammatory phenotype while enhancing their ability to recruit and activate other immune cells, including cytotoxic T cells and natural killer (NK) cells [348].

Lastly, biomaterial-based delivery systems can help insulate CAR-Ms from TME-derived signals. Encapsulation in immune-modulating hydrogels or nanoparticle-mediated delivery of small-molecule activators can create a localised microenvironment that supports CAR-M function while shielding them from suppressive factors [349, 350]. By implementing these strategies, CAR-M therapy can overcome the challenge of macrophage plasticity, ensuring sustained anti-tumour activity and reducing the risk of immunosuppressive reprogramming within the hostile TME.

##### Gene transfer

The difficulty in gene transfer to macrophages presents a significant challenge in CAR-M therapy, as these cells are inherently resistant to viral transduction, making gene editing both costly and technically demanding [351]. Several strategies have been developed to enhance gene delivery efficiency (Fig. 6D). Non-viral gene delivery methods, such as electroporation, mRNA transfection, and lipid nanoparticle-based approaches, offer alternatives to traditional viral vectors, reducing safety concerns and improving transfection rates [352]. Additionally, adenoviral vectors specifically designed for myeloid cell transduction have demonstrated enhanced gene transfer efficiency in macrophages [353]. A pivotal study by Klichinsky et al. demonstrated that human macrophages, genetically modified with a CAR via a chimeric adenoviral vector (Ad5f35), not only acquired antigen-specific phagocytic activity but also adopted a stable pro-inflammatory M1 phenotype. In vitro, CAR-Ms exhibited effective tumour cell clearance and secreted pro-inflammatory cytokines and chemokines. In vivo, a single CAR-M infusion significantly reduced tumour burden and prolonged survival in two xenograft mouse models of solid cancer. Importantly, these CAR-Ms also reprogrammed the immunosuppressive TME by converting bystander M2 macrophages to M1, enhancing antigen presentation, and stimulating endogenous T cell responses [278].

Another seminal work demonstrated a novel in vivo programming strategy to generate CAR-M1 macrophages capable of targeting solid tumours without the need for ex vivo manipulation. In this study, nanocomplexes composed of macrophage-targeting carriers and plasmid DNA encoding a CAR-interferon-γ construct were systemically delivered, resulting in the in situ generation of CAR-M1 macrophages with enhanced phagocytic and immunomodulatory functions. These in vivo–engineered CAR-Ms exhibited robust tumour infiltration, antigen-specific phagocytosis, and the ability to modulate the immunosuppressive microenvironment, ultimately leading to significant inhibition of solid tumour growth [354].

Another promising approach is biomaterial-assisted gene delivery, which utilises engineered carriers, such as hydrogels or polymer-based nanoparticles, to facilitate in situ transfection and stable CAR gene integration in macrophages [349, 350]. These strategies collectively aim to improve the feasibility and efficiency of CAR-M engineering, advancing their therapeutic potential in solid tumour immunotherapy.

##### Off-target effects

Excessive macrophage activation can trigger cytokine storms and systemic toxicity, posing a serious challenge in CAR-M therapy (Fig. 6E). Tunable CAR constructs utilised in CAR-T cells, such as drug-inducible or synNotch systems, allow controlled macrophage activation, reducing unintended immune responses [355]. Regulating cytokine secretion through RNA interference (RNAi) or synthetic regulatory circuits can further prevent excessive inflammatory signalling [356]. Safety mechanisms like suicide switches, including inducible caspase-9 or rapamycin-based apoptosis triggers, enable rapid elimination of CAR-Ms in case of severe toxicity [357]. Local delivery strategies, such as intratumoral injections or hydrogel-based CAR-M deployment, help confine therapeutic effects to the tumour site, minimising systemic side effects [358]. These approaches enhance the safety of CAR-M therapy while maintaining its anti-tumour efficacy.

##### Limited antigen presentation

CAR-Ms exhibit weaker antigen presentation than dendritic cells, limiting their ability to trigger strong adaptive immune responses. Several strategies can enhance their antigen-presenting capabilities (Fig. 6F). Engineering CAR-Ms to overexpress MHC-II molecules and co-stimulatory ligands like CD80 and CD86 can improve T cell activation, strengthening anti-tumour immunity [359]. Another approach involves creating macrophage-dendritic cell hybrids (CAR-M/DCs) that combine the phagocytic abilities of macrophages with the superior antigen-presenting function of dendritic cells, enabling enhanced cross-presentation [360]. Additionally, boosting the release of CAR-M-derived exosomes loaded with tumour antigens may improve T cell cross-priming, extending the immune response beyond direct CAR-M activity [361]. These strategies can help bridge the innate and adaptive immunity gap, making CAR-M therapy more effective against solid tumours. Addressing these limitations requires advancements in gene editing, biomaterial-supported delivery, and combinatorial treatment approaches that can enhance CAR-M efficacy in solid tumours. Figure 6 illustrates the key challenges associated with CAR-Ms and strategic approaches to enhance their targeting, persistence, and safety.

### Gamma delta CAR-T cells

γδ CAR-T cells offer a compelling advancement in cancer immunotherapy by combining innate and adaptive immune functions with MHC-independent tumour recognition. This enables them to target a broad range of malignancies, including solid tumours that often evade αβ CAR-T cells through MHC downregulation [268]​.

Their dual cytotoxicity, mediated via CARs and the endogenous γδ TCR, allows recognition of both antigen-specific and stress-induced ligands, increasing tumour-targeting precision [269]. Additionally, γδ CAR-T cells modulate the TME by secreting IFN-γ and TNF-α, activating DCs, NK cells, and macrophages. They also mediate ADCC through Fc receptor interactions, further enhancing their cytolytic potential [270].

Moreover, γδ T cells exhibit enhanced infiltration into solid tumours, a major limitation for αβ CAR-T cells due to the immunosuppressive TME [362, 363] (Table 4). Their low risk of inducing GVHD supports the development of allogeneic, off-the-shelf γδ CAR-T therapies derived from healthy donors, enabling scalable and rapid clinical deployment [279].

In preclinical studies, γδ CAR-T cells targeting CD19, CD123, and CD20 have shown potent anti-tumour activity in leukaemia and lymphoma models, with significant tumour burden reduction in vivo [364–366]​. In solid tumours, γδ T cells have shown promising infiltration and cytotoxicity against hepatocellular carcinoma and glioblastoma models, indicating their potential as a viable alternative to αβ CAR-T cells​ [283, 283].

Clinical trials validating the therapeutic potential of γδ T cells are initiated. A phase I trial evaluating CD20-targeted Vδ1 + CAR-T cells (NCT04735471) in B-cell malignancies [367]​. Additionally, allogeneic γδ T cell (ADI-001) transfer in patients with R/R AML has been well tolerated, with no major safety concerns and encouraging initial response rates​ [280, 368]. Despite their advantages, challenges remain in optimising γδ CAR-T-cell persistence, expansion, and tumour homing in vivo [269]. Altogether, γδ CAR-T cells offer MHC-independent targeting, low GVHD risk, strong cytotoxicity, and allogeneic applicability, making them a promising immunotherapy.

### CAR-natural killer T cells

CAR-NKT cells represent a novel immunotherapeutic platform that combines the cytotoxic capacity of CAR-T cells with the innate immunity of NKT cells. Unlike CAR-T cells, CAR-NKT cells function independently of MHC, reducing GVHD risk and enabling universal, allogeneic applications. Li et al. generated universal CAR-NKT (UCAR-NKT) cells from HSCs with TCR engineering and HLA gene editing, which exhibited high purity, resistance to allorejection, and potent anti-tumour activity against blood and solid cancers [271, 272].

CAR-NKT cells outperform both CAR-T and CAR-NK therapies in several key areas: superior tumour infiltration, enhanced persistence through memory subsets, and dual tumour recognition via CAR and NKT receptors. Their controlled persistence mitigates risks such as CRS and “on-target, off-tumour” toxicity often seen with CAR-T cells ​[273].

Preclinical studies show robust efficacy against various tumours. GD2-CAR-NKT cells suppressed neuroblastoma with high infiltration and minimal toxicity [286]​. CD19-targeting CAR-NKT cells outperformed conventional CAR-T cells in B-cell lymphoma models, showing improved tumour clearance, persistence, and immune modulation​ [288]. In melanoma models, CAR-NKT cells targeting chondroitin sulfate proteoglycan 4 (CSPG4) demonstrated superior tumour elimination compared to CAR-T cells, with faster expansion rates and enhanced infiltration into TME [275]​.

Several clinical trials are underway to assess the safety and efficacy of CAR-NKT cells in cancer therapy. A phase I trial (NCT03294954) investigating GD2-CAR-NKT cells in children with neuroblastoma demonstrated promising outcomes, including significant tumour regression with no CRS or neurotoxicity ​[276]. Another ongoing trial (NCT04814004) evaluates CD19-directed CAR-NKT cells expressing IL-15 in relapsed/refractory B-cell malignancies​ [273]. Notably, CAR-NKT cells in these trials have shown improved tumour localisation and persistence while avoiding severe immune-related adverse events. CAR-NKT cells thus offer a highly effective, low-toxicity alternative in both haematological and solid malignancies, warranting further clinical validation. Table 4 highlights the key differences between CAR-T, CAR-NK, CAR-M, γδ CAR-T, and CAR-NKT Cell therapies.

## Combination therapies

### CAR-Cells and chemotherapy

Chemotherapy can enhance CAR-cell therapy through several mechanisms. Firstly, it induces lymphodepletion before CAR-T cell infusion. Cyclophosphamide and fludarabine are commonly used to eliminate endogenous immune cells, creating a favourable niche for CAR-T expansion and persistence. Lymphodepletion reduces Tregs and MDSCs, which are known to suppress CAR-T activity. Studies have demonstrated that conditioning with cyclophosphamide/fludarabine enhances CAR-T engraftment, improving response rates in haematological malignancies such as leukaemia and lymphoma​ [369]​. Agents like gemcitabine and paclitaxel have been shown to selectively deplete Tregs and MDSCs while increasing infiltration of cytotoxic T and NK cells​ [370]. Anthracyclines and alkylating agents have been shown to increase the expression of TAAs and disrupt protective tumour stroma, thereby sensitising tumour cells and enhancing immune-mediated tumour clearance​ [371]. Nab-paclitaxel and docetaxel have been found to degrade fibrotic stroma, enhancing CAR-cell access to tumour nests​ [372]. Doxorubicin downregulates immune checkpoint molecules such as PD-L1 on tumour cells, improving CAR-T function​ [373].

Clinical trials evaluate the combination of chemotherapy and CAR-T cell therapy [374]. In a Phase I clinical trial, a combination of Claudin18.2-CAR-T cells with paclitaxel and cyclophosphamide (Cy) was tested in 28 patients who had previously failed taxane-based treatments. The combination resulted in significant clinical responses in 21 patients. The authors suggested that the therapeutic effect may be due to the accumulation of nab-paclitaxel in the tumour stroma, which disrupts cancer-stromal interactions and enhances CAR-T cell infiltration, thus improving the overall efficacy of the treatment [375]. Combining chemotherapy with CAR-cell therapies offers the potential for improved efficacy in solid tumours. Optimising chemotherapy dosing and scheduling to balance tumour reduction with immune preservation and minimising toxicity are required to maximise the treatment success rates.

### CAR-cells and radiotherapy

Radiotherapy (RT) has long been crucial in cancer treatment, and its combination with CAR-T cell therapy is emerging as a promising strategy to overcome key barriers in solid tumours. One of the primary mechanisms by which RT benefits CAR-T therapy is its ability to induce DNA damage in tumour cells, leading to the release of TAAs and neoantigens. Additionally, RT alters the expression of adhesion molecules such as ICAM-1 and VCAM-1, facilitating immune cell infiltration and enhancing CAR-T cell homing to the tumour site​ [376]. Beyond improving antigen presentation, RT can also remodel the TME to create a more favourable environment for CAR-T cell activity. RT can counteract the immunosuppressive TME by normalising the tumour vasculature, reducing hypoxia, and decreasing the presence of immunosuppressive cells [377]. Furthermore, RT has been shown to enhance the production of pro-inflammatory cytokines such as IFN-γ, TNF-α, and IL-12, which promote CAR-T cell activation and persistence.

Preclinical and clinical studies support the synergistic effects of combining RT with CAR-T therapy. For instance, studies have demonstrated that preconditioning tumours with RT before CAR-T cell infusion leads to greater T cell expansion and improved tumour control. In glioblastoma models, RT has been shown to enhance GD2-CAR-T cell infiltration and efficacy, while in pancreatic cancer, low-dose RT has improved CAR-T persistence and cytotoxicity [378, 379]. Clinically, early trials investigating the combination of radiotherapy with CAR-T cell therapy have reported increased tumour regression and prolonged survival compared to CAR-T monotherapy [380, 381]. However, challenges remain, including determining the optimal RT dose and timing relative to CAR-T cell infusion. High-dose RT can cause excessive immune suppression and potentially impair CAR-T function, whereas lower fractionated doses appear more immunogenic. Future research should focus on refining RT-CAR-T combination protocols and integrating additional immunomodulatory agents to maximise therapeutic benefit​ [382].

### CAR-cells and antibodies

Therapeutic antibodies have revolutionised cancer treatment by targeting TAAs with high specificity. These include monoclonal antibodies (mAbs), bispecific T-cell engagers (BiTEs), and antibody–drug conjugates (ADCs). mAbs have demonstrated efficacy across various malignancies, improving survival rates in breast, colorectal, and lymphoma cancers [383]. Bispecific antibodies (BsAbs), such as Bi-specific T-cell engagers (BiTEs), bridge T cells to tumour cells by targeting CD3 on T cells and tumour-associated antigens, thereby facilitating tumour cell elimination independent of MHC restriction [384]. Antibody–drug conjugates (ADCs) consist of a monoclonal antibody linked to a cytotoxic payload via a cleavable or non-cleavable linker, enabling the selective delivery of chemotherapeutic agents to tumour cells while minimising off-target toxicity [385–387].

Studies have frequently demonstrated synergistic effects of combining CAR-T cell therapy with immune checkpoint inhibitors (ICIs), such as anti–PD-1/PD-L1 antibodies, but the clinical translation of these promising preclinical findings into solid tumour settings has been inconsistent. For example, in a phase I trial of intrapleural mesothelin-targeted CAR-T cells involving 27 patients (25 with malignant pleural mesothelioma, MPM), treatment was safe, and CAR-T cells persisted in peripheral blood for over 100 days in 39% of patients. Eighteen patients also received pembrolizumab (anti–PD-1), resulting in a median overall survival of 23.9 months and an 83% one-year survival rate. Additionally, eight patients maintained stable disease for ≥ 6 months, and two exhibited complete metabolic responses, suggesting potential benefit from combining CAR-T cells with PD-1 blockade [388]. In contrast, other studies have demonstrated limited or no survival benefit of ICIs in certain solid tumours with low PD-L1 expression. A pooled analysis of multiple randomized clinical trials in advanced esophageal squamous cell carcinoma revealed no significant overall survival improvement with ICI-based regimens compared to chemotherapy alone in patients with a tumour proportion score (TPS) below 1% (HR = 0.91; 95% CI: 0.74–1.12; P = 0.38) [389]. Similarly, a trial involving 11 patients with relapsed or refractory neuroblastoma compared treatment with CAR-T cells alone (n = 4), CAR-T cells plus cyclophosphamide and fludarabine (Cy/Flu; n = 4), or CAR-T cells combined with Cy/Flu and pembrolizumab (n = 3). While Cy/Flu enhanced CAR-T cell expansion, the addition of pembrolizumab did not improve T-cell accumulation or persistence; only one patient in the pembrolizumab cohort achieved a complete response [390]. Likewise, a phase I trial (NCT03726515) investigating CAR-T cells targeting EGFRvIII combined with pembrolizumab in seven patients with glioblastoma multiforme (GBM) showed no notable efficacy [391].

Studies have demonstrated that BiTE-secreting CAR-T cells can overcome antigen escape and enhance tumour cell killing. For instance, a study utilising EGFRvIII-CAR-T cells engineered to secrete EGFR-specific BiTEs demonstrated enhanced elimination of heterogeneous glioblastoma cells in murine models. BiTE-secreting CAR-T cells did not induce toxicity in human skin graft models [392].

ADCs could help overcome CAR-T limitations in solid tumours by targeting antigen-low cells and modulating the TME. A study combining ADCs and CAR-T cells showed that ADCs tagged with benzylguanine (BG) redirected SNAP-CAR-T cells to tumour antigens. This approach resulted in enhanced tumour cell killing and bystander effects, demonstrating a promising synergy between ADCs and CAR T-cell therapy to address limitations such as inefficient drug delivery and immune evasion in resistant cancers [393]. In CAR-NK therapy, ADCs enhance tumour killing and induce immunogenic cell death, while in CAR-M, they could promote phagocytosis and anti-tumour polarisation [394]. These findings underscore the potential of integrating therapeutic antibodies with CAR-Cell therapies to enhance antigen targeting, mitigate immune escape, and improve clinical outcomes in solid tumour immunotherapy. However, further optimisation is needed to address the limitations observed and enhance the efficacy of this combined approach.

### CAR-cells with small molecule inhibitors

Small-molecule inhibitors (SMIs) are low-molecular-weight compounds that modulate biological processes by targeting specific proteins, pathways, or cellular mechanisms. Unlike monoclonal antibodies or cellular therapies, small molecules possess superior tissue penetration, oral bioavailability, and cost-effectiveness, making them attractive candidates for combination therapies​ [395].

The efficacy of SMIs is well-documented in various cancers, with numerous agents approved for clinical use. For example, tyrosine kinase inhibitors (TKIs), such as imatinib and dasatinib, have demonstrated remarkable success in CML. The EGFR inhibitors (e.g., gefitinib, osimertinib) have significantly prolonged survival in NSCLC​ [396–399]. Combining CAR-Cell therapies with small-molecule inhibitors is emerging as a promising strategy to enhance therapeutic efficacy and overcome resistance mechanisms. For instance, lenalidomide has been shown to enhance CAR-T cell function by increasing the production of key effector cytokines, mitigating T-cell exhaustion and promoting the development of memory phenotypes. For instance, lenalidomide preferentially expanded the CD8 + CAR-T cell subset, resulting in increased total T-cell expansion and maintenance of memory T-cell signatures such as CD62L, CD127, CD27, and CD28 while reducing the expression of exhaustion markers like PD1 and Tim3 [400].

In addition, SMIs sensitise tumour cells. In a study, gilteritinib upregulated the expression of NKG2D ligands on AML cell lines, thereby enhancing the efficacy of FLT3scFv/NKG2D-CAR-T cells. The combination of gilteritinib and these CAR-T cells resulted in synergistic anti-tumour effects, both in vitro and in vivo, against FLT3-mutated AML [401]. Furthermore, small molecules can modulate the TME to optimise CAR-T function. Dasatinib, an Lck inhibitor, is pivotal in preventing excessive CAR-T activation, which can lead to severe CRS [402].

SMIs are also emerging to enhance the activity of CAR-NK cells and CAR-Ms. For instance, proteasome inhibitors, such as bortezomib, have been shown to upregulate tumour antigen expression, thereby enhancing CAR-NK cell-mediated killing [403]. Histone deacetylase inhibitors (HDACi) and MEK inhibitors have been shown to shift macrophages toward a pro-inflammatory, tumouricidal phenotype, while TGF-β inhibitors help counteract MDSC-mediated immune evasion [404–406]. Despite their potential, SMIs face challenges like drug resistance, off-target toxicity, short half-life, and poor TME penetration in solid tumours. Optimising drug selectivity, overcoming resistance, and refining combination strategies will be crucial for maximising their therapeutic impact [407].

### CAR-cells with cancer vaccines

Cancer vaccines enhance antitumour immunity by stimulating the adaptive immune system to recognise and eliminate malignant cells. Cancer vaccines introduce TAAs or neoantigens to activate APCs like DCs and macrophages. These present antigens via MHC molecules, triggering cytotoxic CD8 + T cells to lyse tumour cells and helper CD4 + T cells to enhance immune responses. Memory T cells ensure long-term protection against recurrence. Additionally, cancer vaccines modulate the TME by reducing immunosuppressive cells (Tregs, MDSCs) and increasing pro-inflammatory cytokines (IL-12, IFN-γ), creating a more immunogenic environment [408].

Cancer vaccines have been explored to optimise antigen presentation and immune activation. mRNA-based cancer vaccines, which use lipid nanoparticle (LNP)-encapsulated mRNA encoding tumour antigens, can efficiently transfect DCs and enhance antigen presentation [409]. Peptide-based vaccines, composed of synthetic peptides derived from TAAs, stimulate both CD8 + and CD4 + T cell responses upon uptake by APCs [410]. Dendritic cell-based vaccines involve autologous DCs pulsed with tumour antigens ex vivo before reinfusion [411]. Viral vector-based vaccines, such as adenovirus or vaccinia virus vectors, efficiently deliver tumour antigen genes to APCs [412].

Cancer vaccine-CAR-T cell combination is emerging as a promising strategy to overcome the key limitations of CAR-T therapy in solid tumours. BioNTech’s BNT211 combines an mRNA vaccine with CAR-T therapy to target Claudin 6 (CLDN6), an oncofetal antigen in solid tumours. Preclinical ovarian and testicular cancer models showed that the CLDN6 mRNA vaccine enhanced CAR-T expansion, persistence, and cytokine production, improving tumour regression. The Phase 1/2 BNT211-01 trial, combining CLDN6-CAR-T cells with the CAR-T cell-amplifying RNA vaccine (CARVac) in relapsed/refractory CLDN6-positive solid tumours, reported robust CAR-T engraftment with manageable toxicity (46% CRS, 5% mild neurotoxicity). The ORR was 33%, DCR was 67%, and germ cell cancer patients who were given a higher dose had the best outcomes (ORR 57%). These results highlight the potential of mRNA vaccines to enhance CAR-T efficacy in solid tumours [413]. Another Phase 1 trial of NY-ESO-1-specific TCR-T cells with a pullulan nanogel: LPA vaccine in advanced soft tissue sarcoma showed enhanced TCR-T expansion and CXCR3 expression without lymphodepletion. Three HLA-matched patients with refractory synovial sarcoma received two TCR-T infusions with vaccination before and after treatment. Two had mild-to-moderate CRS, and one showed significant tumour shrinkage lasting over two years with long-term TCR-T persistence, demonstrating the feasibility and durable therapeutic potential (JMA-IIA00346) [414]. Cancer vaccines enhance CAR-T therapy but require optimised timing, dosing, and toxicity management. Personalised neoantigen vaccines are promising strategies to improve solid tumour immunotherapy [408].

### CAR-cells with oncolytic viruses

Oncolytic viruses (OVs) are a novel cancer therapy that selectively infects and lyses tumour cells. Genetically engineered or naturally occurring, OVs improve CAR-T cell efficacy by directly causing oncolysis, releasing tumour antigens and pro-inflammatory signals that trigger immunogenic cell death. Additionally, OVs remodel the TME by reducing immunosuppressive cells like Tregs and MDSCs while promoting immune-stimulatory molecules such as GM-CSF, IL-12, and IFN-β to enhance CAR-T activation and persistence. OVs secrete chemokines, such as CXCL9 and CXCL10, which attract CAR-T cells into the tumour core, thereby enhancing T cell homing into the tumour site. Moreover, OV-induced lysis exposes TAAs. [415].

Studies have demonstrated the potential of OV and CAR-T cell combinations in cancer. In a study, the combination of an EGFR-targeting oncolytic adenovirus (Onc.Ad-EGFR BITE) with folate receptor-alpha (FR-α)-specific CAR-T cells demonstrated improved tumour cell killing through the secretion of bispecific T-cell engagers (BITE) in pancreatic and colorectal carcinoma [416]. Similarly, in neuroblastoma, an oncolytic adenovirus expressing RANTES and IL-15 in combination with GD2-specific CAR-T cells significantly improved CAR-T cell trafficking, survival, and anti-tumour efficacy [417]. Additionally, OV-loaded CAR-T cells have been explored as a novel strategy to deliver oncolytic viruses directly to tumour sites. One study demonstrated that CAR-T cells successfully transferred OVs into tumour cells, leading to enhanced viral replication, oncolysis, and increased CAR-T infiltration [418].

Clinically, OV-CAR-T combinations are still in the early stages. Talimogene laherparepvec (T-VEC), an oncolytic herpes simplex virus expressing GM-CSF, has been FDA-approved for melanoma and is being investigated for combination with CAR-T therapy [419]. Moreover, another clinical trial (NCT03740256) is currently evaluating HER2-specific CAR-T cells in combination with an oncolytic adenovirus in HER2-positive solid tumours [420]. These findings highlight the growing interest in OV-CAR-T strategies and their potential to improve therapeutic outcomes by overcoming key barriers associated with solid tumours.

### Multi-CAR-cell combination therapy

**Fig. 7:**
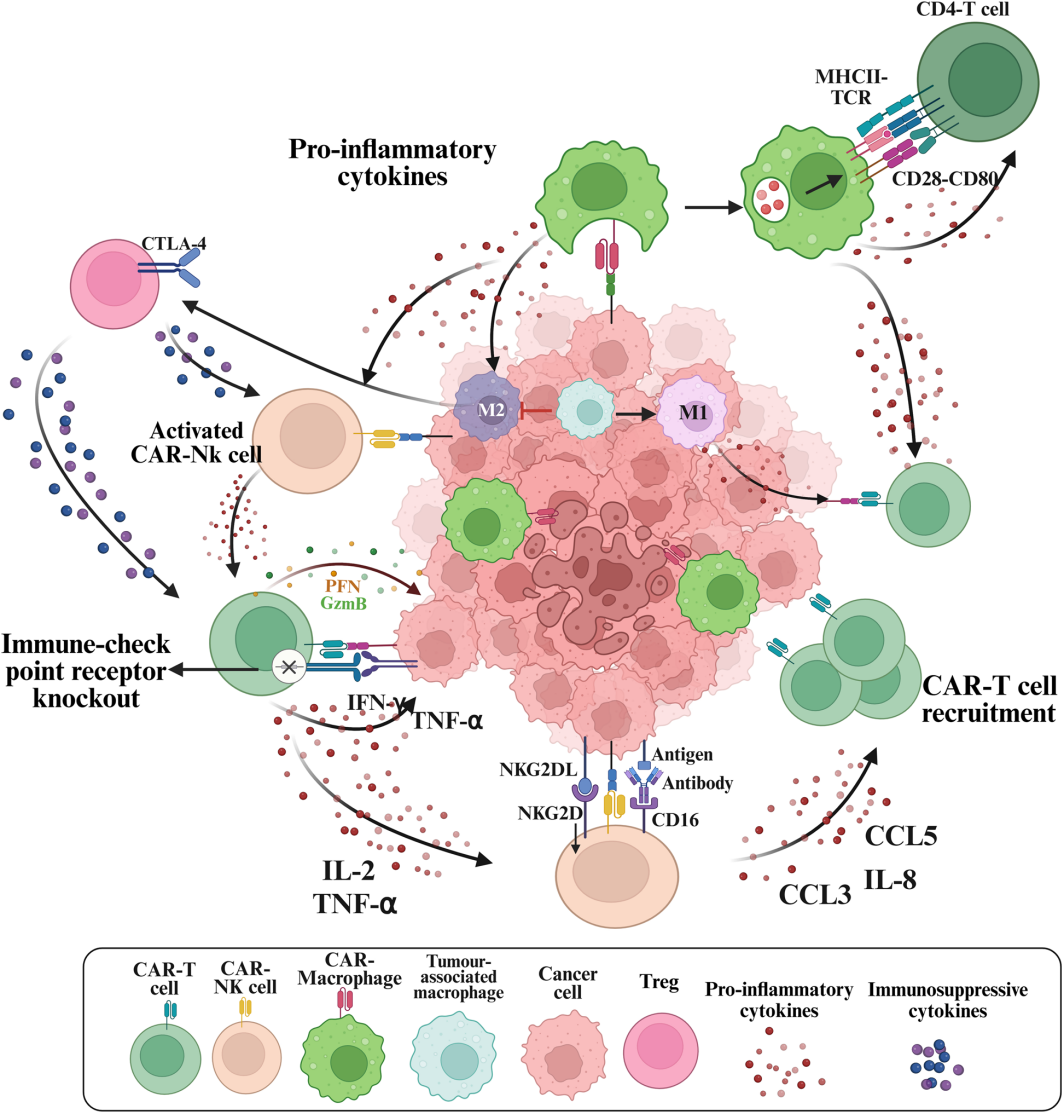
The multi-CAR-cell combination strategy harnesses the complementary functions of CAR-T cells, CAR-NK cells, and CAR-Ms to overcome the complex barriers presented by solid tumours. CAR-T cells, genetically modified to target TAAs, are central to the cytotoxic response. Once activated, they proliferate, secrete effector molecules, and directly lyse tumour cells. However, in the context of solid tumours, CAR-T cells face several limitations, including poor infiltration into the TME, antigen heterogeneity, and functional exhaustion driven by chronic stimulation and immunosuppressive cues. To enhance their activity, CAR-NK cells are co-administered. These cells exhibit rapid, antigen-independent killing via NKG2D–NKG2DL interactions and CD16-mediated ADCC, and importantly, they secrete cytokines such as IFN-γ and TNF-α, which stimulate both CAR-T cells and innate immunity. Their presence helps remodel the TME and improves CAR-T cell recruitment and function. CAR-Ms further support this cooperative network. By infiltrating the tumour mass, phagocytosing cancer cells, and shifting towards an M1-like phenotype, they promote inflammation, antigen presentation, and T-cell recruitment. Through secretion of chemokines and cytokines, including IL-12 and CCL3/CCL5, CAR-Ms enhance T cell infiltration and polarise the TME in favour of anti-tumour immunity. Collectively, these three engineered cell types reinforce one another. CAR-NKs and CAR-Ms condition the TME to support deeper CAR-T cell penetration and persistence. Immune-checkpoint receptor knockouts further sustain activity by resisting TME-induced inhibition. This tripartite approach amplifies immune activation, promotes tumour clearance, and represents a promising avenue to overcome the immunosuppressive nature of solid tumours

The multi-CAR-cell combination therapy represents a promising approach for overcoming the limitations of individual CAR-based therapies in solid tumours (Fig. 7). CAR-T cells, CAR-NK cells, and CAR-Ms each possess distinct advantages that, when integrated, create a synergistic and multifaceted immune response against tumours. CAR-T cells exhibit strong antigen-specific cytotoxicity and long-term persistence, but they struggle with tumour infiltration and are prone to exhaustion within the immunosuppressive TME [421]. In contrast, CAR-NK cells, with their innate cytotoxicity and ability to mediate ADCC, provide an additional tumour-killing mechanism that is less reliant on antigen specificity, making them effective against heterogeneous tumour populations and enabling them to eliminate tumour cells that may have downregulated their antigen expression to escape CAR-T cell therapy [422]. Furthermore, CAR-Ms serve as powerful modulators of the TME, actively infiltrating solid tumours, reshaping the immune landscape, and enhancing the activity of both CAR-T and CAR-NK cells through cytokine secretion and antigen presentation [423].

The combination of CAR-T and CAR-NK cells is particularly effective in solid tumours due to their complementary mechanisms of action. While CAR-T cells target specific TAAs, CAR-NK cells provide broader recognition through activating receptors such as NKG2D, which detect stress-induced ligands commonly upregulated in tumours. CAR-NK cells also prime the tumour site by secreting chemokines such as CCL3, CCL5, and IL-8, which recruit CAR-T cells and improve their infiltration into the TME [261]. Once activated, CAR-T cells, in turn, secrete pro-inflammatory cytokines such as IL-2 and IFN-γ, further amplifying NK cell activation and persistence. This interplay sustains a prolonged immune response and reduces the likelihood of tumour recurrence.

Additionally, the effectiveness of CAR-T and CAR-NK cells can be further enhanced by equipping them with specific modifications. For example, the co-expression of IL-15 and IL-21 enhances survival, proliferation, and functionality, while engineering these cells to express chemokine receptors such as CXCR3 and CCR5 improves their tumour-homing ability. Furthermore, checkpoint-resistant CAR designs that block PD-1 or CTLA-4 signalling prevent exhaustion, enhancing overall cytotoxicity and persistence within the TME [255, 258, 265].

CAR-Ms provide another critical component in this multi-CAR strategy by actively infiltrating solid tumours and reprogramming the TME to support anti-tumour immunity. Unlike CAR-T and CAR-NK cells, which primarily rely on direct cytotoxicity, CAR-Ms function as APCs, facilitating a broader immune response. Macrophages are naturally attracted to hypoxic and necrotic tumour regions, making them ideal carriers for delivering immune-modulating effects within the TME. However, TAMs are often polarised toward an immunosuppressive (M2) phenotype, which supports tumour growth rather than elimination. Engineering macrophages to express CARs enables them to be reprogrammed into a pro-inflammatory (M1) phenotype, enhancing their phagocytic activity and ability to present tumour antigens to T cells [266, 324].

CAR-Ms support CAR-T and CAR-NK cells by secreting cytokines such as IL-12 and TNF-α, which promote T cell expansion and NK cell activation, thereby increasing overall immune activity within the tumour site. Moreover, the effectiveness of CAR-Ms can be optimised by equipping them with special molecules such as CD40 ligand (CD40L) to enhance T-cell priming, FcγR to improve antibody-dependent cellular phagocytosis (ADCP), and checkpoint inhibitors that prevent macrophage suppression within the TME. By resisting immunosuppressive factors such as TGF-β and IL-10, CAR-Ms maintain their tumouricidal function, ensuring sustained immune activation [424, 425].

The combination of these immune cells creates a dynamic anti-tumour response where macrophages serve as the initial infiltrators, remodelling the TME by secreting pro-inflammatory cytokines that attract and enhance the function of CAR-T and CAR-NK cells. Integrating CAR-T, CAR-NK, and CAR-Ms into a unified therapeutic strategy maximises the advantages of each cell type while addressing key challenges associated with solid tumour treatment (Fig. 7 and Table 5).

**Table 5:** Summary of CAR-Cell Combination Therapeutic Strategies in Solid Tumours

Combination Strategy	Mechanism of Action	Therapeutic Outcomes	References
CAR-Cells + Chemotherapy	• Induces lymphodepletion to create a niche for CAR-cell expansion • Depletes immunosuppressive cells (Tregs, MDSCs) • Increases tumour antigen expression • Disrupts stroma to enhance CAR-cell infiltration	Improved CAR-cell engraftment and persistence; enhanced tumour infiltration; significant clinical responses in solid tumours	[369–375]
CAR-Cells + Radiotherapy	• Induces DNA damage, releasing TAAs and neoantigens • Upregulates adhesion molecules (ICAM-1, VCAM-1) to enhance CAR-cell homing • Remodels tumour microenvironment (normalises vasculature, reduces hypoxia and immunosuppressive cells) • Promotes pro-inflammatory cytokine production (IFN-γ, TNF-α, IL-12)	Synergistic tumour regression; enhanced CAR-cell infiltration, expansion, persistence; improved survival in preclinical and clinical models	[378–382]
CAR-Cells + Therapeutic Antibodies	• Targets TAAs or immune checkpoints via monoclonal antibodies • Uses BiTE-secreting CARs to overcome antigen escape • Employs ADCs for selective cytotoxic delivery and TME modulation	Enhanced tumour killing; reversal of CAR-cell exhaustion; improved antigen coverage; clinical trials show variable efficacy; potential synergy demonstrated preclinically	[388–394]
CAR-Cells + SMIs	• Targets signalling pathways to reduce immunosuppression (e.g., TGF-β, JAK/STAT) • Sensitises tumour cells and reduces CAR-cell exhaustion (e.g., TKIs) • Modulates the TME and immune cell phenotypes (e.g., HDAC inhibitors)	Enhanced CAR-cell proliferation, cytotoxicity, and persistence; modulation of TME; potential to reduce CRS severity; challenges include resistance and toxicity	[400–406]
CAR-Cells + Cancer Vaccines	• Enhances antigen presentation and adaptive immune activation via mRNA, peptide, DC-based, or viral vector vaccines • Modulates the TME by reducing suppressive cells and increasing pro-inflammatory cytokines	Improved CAR-cell expansion, persistence, and cytokine production; enhanced tumour regression; promising early-phase clinical results	[408–414]
CAR-Cells + OVs	• Selectively lyses tumour cells, releasing TAAs and pro-inflammatory signals • Remodels tumour microenvironment by reducing suppressive cells and secreting chemokines (CXCL9, CXCL10) • Employs OV-loaded CAR-cells for targeted virus delivery	Increased CAR-cell tumour infiltration, survival, and anti-tumour efficacy; synergistic tumour killing demonstrated preclinically; ongoing clinical evaluation	[415–420]
Multi-CAR-Cell Combination Therapy	• Combines CAR-T, CAR-NK, and CAR-M cells for a multifaceted immune response • CAR-T: antigen-specific cytotoxicity, long persistence; limited infiltration, prone to exhaustion • CAR-NK: innate cytotoxicity, ADCC, broad recognition, secretes chemokines recruiting CAR-T cells • CAR-M: infiltrates tumour, reprograms TME, presents antigen, secretes IL-12 and TNF-α to enhance T/NK cells • Engineering modifications such as novel CAR designs, co-expression of IL-15/IL-21, chemokine receptors (CXCR3, CCR5), and checkpoint-resistant designs (PD-1/CTLA-4 blockade) can significantly improve these CAR-based cell types	Potential outcome: Enhanced tumour clearance through complementary and sustained immune activation; improved infiltration, persistence, and resistance to TME immunosuppression	[255, 258, 261, 265, 266, 324, 421–425]

## Conclusion and perspectives

The advancement of CAR-Cell therapies holds significant potential for transforming the treatment landscape of cancer, yet several challenges remain, including immunosuppressive TME, poor CAR-cell infiltration, antigen heterogeneity, CAR-cell exhaustion, off-target effects, and manufacturing challenges. An increasingly pressing question is which immune effector cell types, CAR configurations, and combination strategies will best fulfil the unmet clinical needs in both haematological and solid malignancies.

CAR-T cells, the most clinically advanced modality, have demonstrated durable remissions in haematological cancers, but their efficacy in solid tumours remains limited by antigen dependence, poor tumour infiltration, and TME-induced dysfunction. Moreover, life-threatening toxicities like CRS and neurotoxicity, along with the logistical complexity of autologous manufacturing, pose significant barriers. Next-generation CAR-T cells equipped with multi-targeting and modular CAR constructs may significantly enhance therapeutic functionality. However, their clinical translation will require rigorous and time-intensive evaluation [138].

CAR-NK cells offer advantages such as innate cytotoxicity, low risk of CRS and GVHD, and compatibility with allogeneic, off-the-shelf production. Despite encouraging early-phase results, limited persistence and suboptimal tumour infiltration currently restrict their standalone efficacy [426].

CAR-Ms represent a novel and highly promising approach for solid tumours. Unlike lymphoid effectors, macrophages naturally traffic into the TME, where they can exert multifaceted functions including antigen-independent phagocytosis, cytokine secretion (e.g., TNF-α, IL-12), and stromal remodelling. Preclinical data and early clinical results from the CT-0508 trial have demonstrated biological activity, T-cell recruitment, and a favourable safety profile in HER2-positive tumours. CAR-Ms’ ability to bypass antigen escape and reprogram immunosuppressive microenvironments positions them as a potentially transformative modality for solid tumour immunotherapy [329, 330]. Furthermore, emerging in vivo CAR engineering strategies now enable the direct reprogramming of macrophages into M1-polarised CAR-Ms within the TME using both viral vectors and non-viral platforms, such as macrophage-targeting nanocomplexes and nanomaterial-based delivery systems [240, 278, 354]. These in situ–engineered CAR-Ms exhibit enhanced tumour-directed phagocytosis and immunomodulatory function, offering a scalable, off-the-shelf therapeutic strategy that avoids the complexities of ex vivo manufacturing. The ability of CAR-Ms to overcome antigen escape and reshape the immunosuppressive TME underscores their transformative potential in solid tumour immunotherapy. However, clinical translation and manufacturing standardisation persist, necessitating further optimisation [36].

Unconventional platforms such as γδ CAR-T and CAR-NKT cells are also under active investigation. γδ CAR-T cells provide dual targeting through CARs and their endogenous TCR, enabling recognition of stress ligands and greater resistance to antigen escape. CAR-NKT cells combine CAR-mediated cytotoxicity with innate immune modulation, supporting dendritic cell and NK cell activation. Both cell types exhibit favourable safety and enhanced tumour infiltration in preclinical models, but remain in early clinical stages with limited translational data. Altogether, while CAR-T cells remain the clinical standard for haematological malignancies, recent innovations in CAR engineering are redefining the therapeutic potential of CAR-T cells, enabling more precise control over their activation, persistence, and safety profiles. These developments may ultimately expand the applicability of CAR-T cells beyond haematological contexts and into solid tumour indications previously deemed refractory. Emerging platforms such as CAR-NK, CAR-M, γδ CAR-T, and CAR-NKT cells offer distinct immunological and translational advantages. Among these, CAR-macrophages stand out for their ability to infiltrate the immune-excluded TME, exert antigen-independent cytotoxicity, secrete pro-inflammatory cytokines, and reshape the tumour stroma, traits particularly suited for tackling solid tumours [427].

CAR constructs have undergone extensive evolution, with advanced designs offering crucial enhancements in specificity, safety, and tumour control, especially in challenging solid tumours. Among multi-target CARs, bicistronic and trivalent CARs improve tumour targeting by recognising multiple antigens, reducing the risk of antigen escape. However, they can introduce signalling imbalances and require careful antigen pairing to avoid off-target effects. Loop CARs and tandem CARs enhance antigen-binding flexibility and affinity, but their structural complexity may impact surface expression and stability. On the modular front, logic-gated CARs (AND, IF-Better, and NOT/iCARs) provide fine-tuned specificity by ensuring activation only in high-antigen-density environments or suppressing responses to healthy tissues, offering clear safety advantages in solid tumours with heterogeneous expression. SUPRA CARs and synNotch systems represent a shift toward programmable and conditionally activated platforms, allowing real-time control over antigen targeting and reducing tonic signalling, though they require rigorous optimisation to balance activation timing and antitumour potency. Avidity CARs, by needing dual low-affinity interactions, increase discrimination against normal cells but may underperform in low-antigen-density tumours [187]. Taken together, no single CAR design is universally superior, but hybrid strategies, such as combining dual-antigen recognition with a suicide switch or pairing SUPRA systems with synNotch logic, hold promise to address the dual challenges of efficacy and safety in solid tumours. Future adaptation should focus on tumour-selective activation, resistance to suppressive TME, and control over cytokine toxicity, possibly through inducible or self-regulating modules integrated into next-gen CAR constructs.

Among the various combination strategies explored to enhance CAR-based therapies for solid tumours, the integration of CAR-Cells with cancer vaccines, oncolytic viruses, and multi-cell CAR approaches currently holds the most significant promise. Each strategy offers unique mechanisms to overcome the major barriers of antigen heterogeneity, TME immunosuppression, and limited CAR-cell persistence [428]. Cancer vaccines, particularly mRNA-based platforms, enhance antigen-specific T cell responses by priming the immune system with tumour-associated or neoantigens, thereby promoting CAR-cell expansion, memory formation, and infiltration into solid tumours. Clinical evidence from early trials such as BNT211, combining Claudin 6-targeted CAR-T cells with an mRNA vaccine, demonstrates robust CAR-T engraftment, durable responses, and manageable toxicity, underscoring their translational potential [413]. Similarly, OVs represent a powerful modality by directly lysing tumour cells, releasing tumour antigens, and secreting immunostimulatory cytokines such as GM-CSF and IL-12 that enhance CAR-T activation and persistence [429]. Engineered OVs can also express BiTEs or chemokines (e.g., CXCL9/10), facilitating T cell recruitment and remodelling the TME to support immune cell infiltration and activity [416]. Though still in early clinical development, studies using OVs in combination with CAR-T cells show enhanced tumour clearance and the potential for systemic anti-tumour immunity. In parallel, multi-CAR-cell strategies that integrate CAR-T, CAR-NK, and CAR-Ms offer a synergistic immune attack by combining the antigen-specific cytotoxicity of CAR-T cells, the innate killing and ADCC capabilities of CAR-NK cells, and the TME-modifying, antigen-presenting function of CAR-Ms. This combinatorial approach enables more robust and durable responses across heterogeneous tumour cell populations and immunosuppressive niches. However, its implementation is technically complex due to the differing biology, expansion requirements, and manufacturing protocols of each cell type, which currently limit its clinical scalability.

In comparison to more established combinations such as chemotherapy, radiotherapy, antibodies, or small molecule inhibitors, which show varying degrees of benefit but are limited by toxicity, inconsistent results, or lack of tumour specificity, cancer vaccines, oncolytic viruses, and multi-cell CAR strategies provide a more targeted and biologically integrated means of enhancing CAR-based therapies. Collectively, these emerging modalities offer a promising frontier for solid tumour immunotherapy, especially as technologies advance to overcome logistical and biological challenges.

The evolution of CAR-based cellular therapies is rapidly accelerating, driven by the need to overcome the persistent limitations encountered in solid tumour treatment. Emerging cell platforms, sophisticated CAR constructs, innovative combination modalities, and advances in in vivo CAR-cell production are collectively redefining what is possible in cancer immunotherapy. Moving forward, a precision-guided approach, matching the most suitable effector cell and CAR design to each tumour type and microenvironment, and integrating it within a biologically rational combination framework, will be critical. As these technologies continue to evolve, the vision of delivering safe, durable, and broadly effective CAR-cell therapies across cancer types appears not only achievable but inevitable.

## Data Availability

No datasets were generated or analysed during the current study.

## Abbreviations

ALL: Acute lymphoblastic leukaemia
CAR: Chimeric antigen receptor
CAR-M: Chimeric antigen receptor-macrophages
CAR-NK: Chimeric antigen receptor-natural killer cells
CAR-NKT: Chimeric antigen receptor-natural killer T cells
CLL: Chronic lymphoid leukaemia
CSF1R: Colony-stimulating factor 1 receptor
CRS: Cytokine release syndrome
CTLA-4: Cytotoxic T-lymphocyte-associated protein 4
CTLs: Cytotoxic T lymphocytes
DLBCL: Diffuse large B-cell lymphoma
ECM: Extracellular matrix
GVHD: Graft-versus-host disease
HIF-1α: Hypoxia-inducible factor 1-alpha
IDO: Indoleamine 2,3-dioxygenase
IFN-γ: Interferon-γ
IL-2Rβ: Interleukin-2 receptor β-chain
IL-7: Interleukin-7
IL-10: Interleukin-10
IL-12: Interleukin-12
IL-15: Interleukin-15
IL-18: Interleukin-18
IL-21: Interleukin-21
iPSC: Induced pluripotent stem cells
JAK: Janus kinase
MAIT: Mucosal-associated invariant T
MCL: Mantle cell lymphoma
MDSCs: Myeloid-derived suppressor cells
MHCs: Major histocompatibility complexes
MM: Multiple myeloma
NHL: Non-Hodgkin lymphoma
NO: Nitric oxide
OXPHOS: Oxidative phosphorylation
PD-L1: Programmed death-ligand 1
R/R: Relapsed/refractory
ROS: Reactive oxygen species
scFv: Single-chain variable fragment
STAT: Signal transducer and activator of transcription
TAAs: Tumour-associated antigens
TAMs: Tumour-associated macrophages
TCR: T-cell receptor
TGF-β: Transforming growth factor-beta
TNF-α: Tumour necrosis factor-alpha
TME: Tumour microenvironment
TRUCKs: T-cells Redirected for Universal Cytokine-mediated Killing
Tregs: Regulatory T cells
TSAs: Tumour-specific antigens
VEGF: Vascular endothelial growth factor
VH: Variable heavy
VL: Variable light
